# Evaluating patient-reported outcomes in randomized controlled trials of targeted therapy and/or immunotherapy for liver cancer: a scoping review

**DOI:** 10.3389/fonc.2026.1770579

**Published:** 2026-05-13

**Authors:** Lu Ren, Yuntong Liu, Shangjin Li, Shujin Yue, Chunxiang Su, Jiaying Wang, Yiyuan Zhang

**Affiliations:** 1 School of Nursing, Beijing University of Chinese Medicine, Beijing, China; 2 Beijing University of Chinese Medicine Collaboration Center of Joanna Briggs Institute, Beijing, China

**Keywords:** immunotherapy, liver cancer, patient-reported outcomes, scoping review, targeted therapy

## Abstract

**Objective:**

To assess the reporting characteristics and quality of patient-reported outcomes (PROs) in randomized controlled trials (RCTs) of targeted therapy and/or immunotherapy for liver cancer.

**Methods:**

We systematically searched six electronic databases for relevant RCTs published in English or Chinese from inception to April 17, 2025. The reporting quality of PROs was assessed using the CONSORT-PRO extension and the CONSORT 2010 guidelines.

**Results:**

We included 64 RCTs with 8,494 participants. Of these, 14 studies were confirmatory studies, 31 studies were exploratory studies and 19 studies were categorized as “Other” studies. 23.4% (15/64) of these RCTs reported PROs as the primary outcome. Twenty-seven patient-reported outcome measures (PROMs) about 17 relevant domains were observed in these studies. The domain with the highest reporting frequency was quality of life (53/64). Forty trials utilized a single PROM, while 24 trials employed multiple PROMs. The EORTC QLQ-C30 was the most often used PROM (25/53). Nineteen trials employed single-domain symptoms or specific functioning PROMs. Eight symptomatic adverse events were reported in less than 30% of studies. The mean score of the CONSORT-PRO adherence was 7.5 (3-14) for confirmatory studies, 5.8 (4-9) for exploratory studies and 6.0 (3-9) for other studies. The adherence to CONSORT 2010 was poor among the included trials. Only 3 trials achieved good reporting quality.

**Conclusion:**

Among the 64 included RCTs, the reporting of specific symptoms and the application of specific PROMs related to targeted and/or immunotherapy were poor. Meanwhile, the integration of PROs with clinical endpoints was suboptimal, and PRO data were seldom used to guide nursing practice. More importantly, the overall adherence to CONSORT-PRO among the included studies was low. Although confirmatory studies demonstrated higher scores than exploratory studies and other types of studies, there was considerable variation within this category. Future studies should prioritize the use of validated, standard disease-specific tools and strengthen the evaluation of treatment-related adverse events. Furthermore, we advocate establishing a collaborative framework that integrates medical, nursing, and patient perspectives. Future studies, particularly confirmatory studies and nursing intervention or supportive care studies, strictly adhere to the CONSORT-PRO guideline.

## 1 Introduction

Liver cancer is a serious public health issue worldwide, with extremely high incidence and mortality rates. It is the sixth most frequently diagnosed cancer and the third leading cause of cancer death (1). The early symptoms of liver cancer are often insidious, but the disease progress is rapid. Consequently, the majority of patients are diagnosed at the intermediate or advanced stage, and have already lost the opportunity for surgical resection (2). For these patients, systemic therapy plays an important role in prolonging survival and improving quality of life (QoL) (3). In systemic therapy, molecular targeted therapy has become a key treatment for liver cancer since 2007 (4–7). Moreover, the programs based on immunotherapy have been acknowledged as the first-line treatment for patients with advanced-stage liver cancer (8). Notably, the first successful research (IMbrave150) in 2020, which combined immunotherapy with targeted therapy, marked the beginning of a new era of combination treatment in liver cancer (7).

In the past, the evaluation of therapeutic efficacy in tumors mainly focused on the objective indicators, such as mortality and the incidence of complications (9). With the application of patient-reported outcomes (PROs), the focus of clinical research has shifted from prolonging patient survival to improving patients’ multidimensional health status (10). PROs are defined as any report on the patient’s health status that come directly from the patient, without interpretation by clinicians or other healthcare providers (11). It complemented the disease-centered outcomes. The International Society for Pharmacoeconomics and Outcomes Research (ISPOR) and the U.S. Food and Drug Administration (FDA) have emphasized that PRO should be an essential metric in evaluating clinical efficacy and reporting clinical trials (12). The assessment of PRO should be integrated into routine clinical assessment (13). Patient-reported outcome measures (PROMs) are standardized instruments used to assess PRO. In clinical practice, PROMs can enable dynamic monitoring of treatment responses and facilitate communication between clinicians and patients (14, 15). Integrating PROMs into routine care is crucial for accurately capturing the disease burden and refining management strategies (16).

However, current evidence indicated that the reporting quality of PRO in randomized controlled trials (RCTs) was suboptimal (17). PRO reports often exhibited differing levels of bias risk (18). To improve the reporting quality of PRO, the International Society for Quality of Life Research (ISOQOL) published the Consolidated Standards of Reporting Trials Extension for Patient-Reported Outcomes (CONSORT-PRO) in 2013 (19). Some researchers have employed this reporting standard to evaluate the reporting quality of PRO in RCTs related to advanced soft tissue sarcoma (20), metastatic colorectal cancer (21), and hematologic malignancies (22). However, these results showed that the reporting quality of PRO items was poor. High-quality PRO reporting is crucial for improving the overall quality of healthcare, which enables clinicians and patients to make wise treatment decisions based on the results of PRO. Moreover, no study has systematically evaluated the reporting quality of PRO in the RCTs of targeted therapy and/or immunotherapy for liver cancer. Therefore, our study aimed to evaluate the reporting quality and application of PRO in RCTs of targeted and/or immunotherapy for liver cancer to provide evidence for the deep implementation of PROs in future research and clinical practice.

## 2 Methods

This review was prepared in accordance with the Preferred Reporting Items for Systematic Reviews and Meta-Analyses extension for scoping reviews (PRISMA-ScR) guidelines (23).

### 2.1 Inclusion criteria and exclusion criteria

The inclusion criteria were as follows: (a) Adult patients diagnosed with liver cancer; (b) Patients treated with targeted therapy and/or immunotherapy; (c) RCTs reporting the results of PROs; (d) Studies published in English or Chinese.

The following studies were excluded: (a) The PROMs were not specified. (b) The full text was unavailable.

### 2.2 Search strategy

A systematic literature search was performed independently by two researchers (R.L. and W.J.Y.) in six electronic databases (PubMed, Embase, Web of Science, CNKI, Wangfang and VIP) from their inception to April 17, 2025. We employed a search strategy that combined Medical Subject Headings (MeSH) and free-text terms to retrieve literature. The complete search strategies for all databases are provided in **Supplementary Appendix 1**.

### 2.3 Data selection

All retrieved studies were imported into EndNote (Version 20.0) for duplicate removal. Two researchers (R.L. and Z.Y.Y.) independently screened studies by reading the title and abstract. For potentially relevant articles, the two reviewers evaluated the full text independently. Any disagreement during the screening process was resolved through discussion or consultation with a third researcher (S.C.X.).

### 2.4 Data extraction

Two reviewers (R.L. and L.S.J.) independently extracted the data by the standardized data extraction form. The extracted data included article title, publication year, study location, treatment regimen, adverse events, PRO, PROMs and so on. Any disagreement was resolved by discussion between the two individuals or consultation with a third researcher (S.C.X.).

### 2.5 The assessment of the reporting quality of PRO

We (R.L. and L.Y.T) used the CONSORT 2010 and CONSORT-PRO checklists to assess the reporting quality of the included trials. CONSORT 2010 has 25 core items, while CONSORT-PRO incorporates 5 supplementary items. CONSORT-PRO is a supplement to the CONSORT 2010, intending to improve the transparency and completeness of PRO reported in RCTs. We independently scored the items of the CONSORT-PRO according to the existing scoring criteria (24, 25). The maximum raw total score of CONSORT-PRO adherence was 15 for trials with a PRO as the primary outcome and 14 for trials with a PRO as the secondary outcome. Finally, we converted raw item adherence scores of the CONSORT-PRO to percentages for further analysis. Adherence was classified as good when the study’s adherence score reached or exceeded 80% of the total score, as moderate when the score ranged from 50% to 79% of the total score, and as poor when the score fell below 50% of the total score (25).

### 2.6 Ethics

Ethical approval was not required for this study as it utilized only publicly available data.

## 3 Results

### 3.1 Study selection

**Figure 1:**
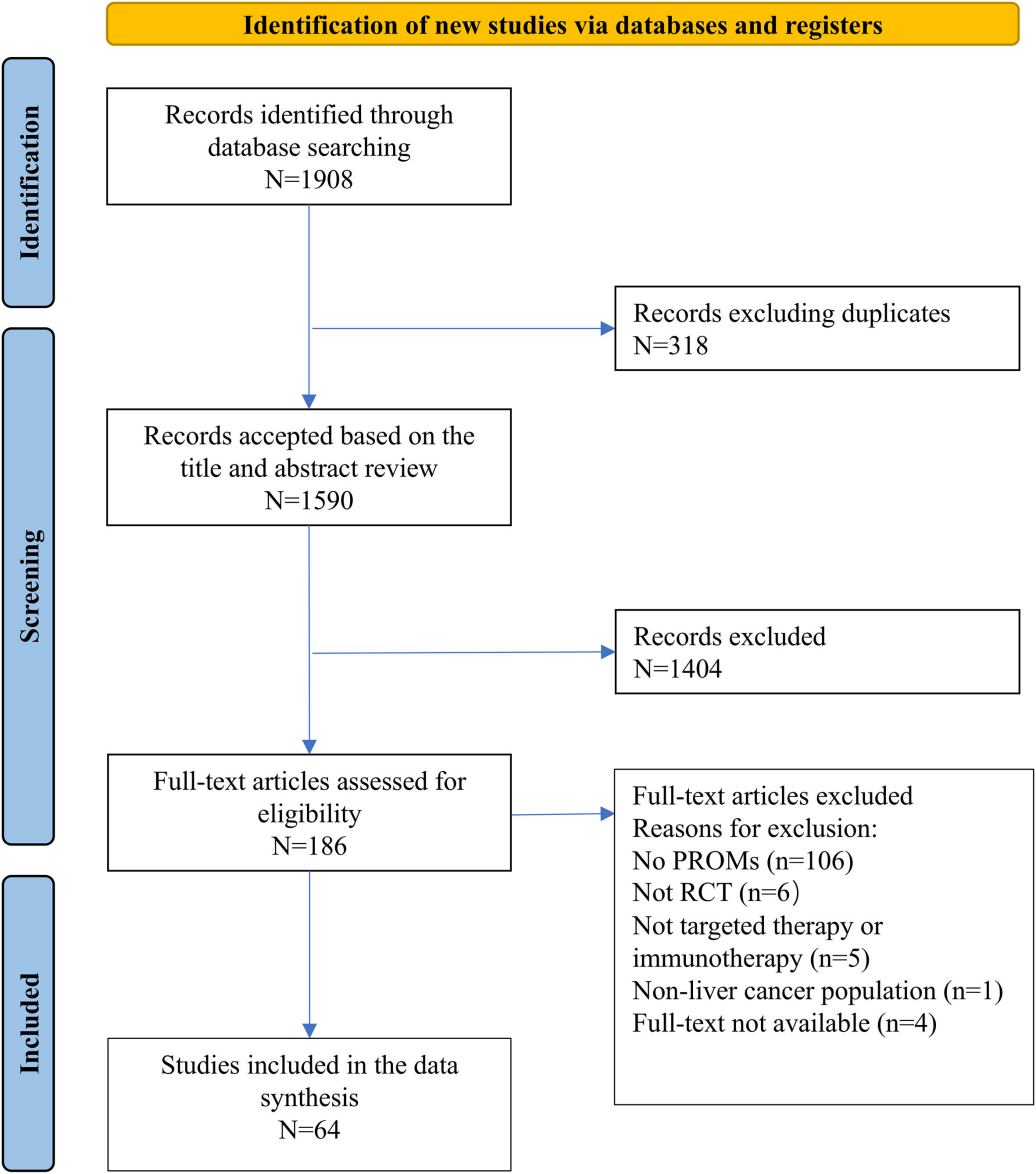
PRISMA flowchart.

A total of 1,908 papers were identified from the initial search. After removing duplicates and articles that did not meet the pre-defined inclusion criteria, 64 studies that reported PROs were included in this review. The selection process of studies was presented in **Figure 1**.

### 3.2 Study characteristics

Sixty-four included trials were published between 2009 and 2025. Fifty studies were conducted in China, while the remaining studies originated from France (n = 4), Spain (n = 2), Germany (n = 2), the United States (n = 2), the United Kingdom (n = 1), Singapore (n = 1), Switzerland (n = 1), and Italy (n = 1). The majority of studies (n = 48) were published in China, the others were published in English. According to the International Council for Harmonization of Technical Requirements for Pharmaceuticals for Human Use (ICH) harmonized guideline E8 (R1) (ICH E8(R1)) framework (26), 14 studies were confirmatory studies (Type C, 21.9%), which were phase III trials or large-scale registration trials intended to support drug licensing or establish definitive efficacy. Thirty-one studies were exploratory studies (Type E, 48.4%), defined as phase I/II trials or small-scale efficacy studies aiming at exploring preliminary efficacy or estimating doses. The remaining 19 studies (29.7%) were categorized as “Other” (Type O), including nursing interventions studies, traditional Chinese medicine studies and supportive care assessment studies that did not meet the conventional staging criteria for clinical trial phases (see **Table 1**).

**Table 1:** Characteristics and key results of included studies (*N* = 64).

First author	Pub. year	Study center	Country	Study type	Sample size/Male	Treatment types	Treatment arms	PROs	PROMs	Outcome	CONSORT-PRO score
A. L. Cheng (61)	2009	Multiple	China	C	226/193	Target therapy VS placebo	Sorafenib VS placebo	Physical well-being	FACT-Hep	SO	6.0
J. Bruix (5)	2017	Multiple	France	C	573/504	Target therapy VS placebo	Regorafenib VS placebo	HRQoL	EQ-5D and EQ-VAS	SO	3.0
T. Meyer (62)	2017	Single	UK	C	313/277	Target therapy plus Chemotherapy VS placebo plus Chemotherapy	Sorafenib plus Chemotherapy VS placebo plus Chemotherapy	QoL	EORTC QLQ-C30 and QLQ-HCC18 and EQ-5D	SO	6.5
V. Vilgrain (63)	2017	Multiple	France	C	459/414	radiation therapy VS Target therapy	Radiation therapy VS Sorafenib	QoL	EORTC QLQ-C30 and QLQ-HCC18	SO	5.5
L. Rimassa (64)	2018	Multiple	Italy	C	340/306	Target therapy VS placebo	Tivantinib VS placebo	QoL	FACT-Hep EQ-5D	SO	4.5
P. K. H. Chow (65)	2018	Multiple	Singapore	C	360/299	radiation therapy VS Target therapy	radiation therapy VS Sorafenib	HRQoL	EQ-5D	SO	8.5
J. L. Jouve (66)	2019	Multiple	France	C	323/298	Target therapy plus lipid lowering drugs VS Target therapy	Sorafenib plus Pravastatin VS Sorafenib	QoL	EORTC QLQ-C30 and FACT-Hep	SO	9.0
R. S. Finn (7)	2020	Multiple	America	C	501/414	Target therapy plus Immunotherapy VS Target therapy	Bevacizumab plus Atezolizumab VS Sorafenib	QoL	EORTC QLQ-C30	SO	4.0
A. Vogel (67)	2021	Multiple	Germany	C	931	Target therapy VS Target therapy	Lenvatinib VS Sorafenib	QoL	EORTC QLQ-C30 and QLQ-HCC18	SO	13.0
B. Y. Ryoo (68)	2021	Single	France	C	410	Immunotherapy VS placebo	Pembrolizumab VS placebo	QoL	EORTC QLQ-C30 and QLQ-HCC18	SO	11.0
P. R. Galle (69)	2021	Multiple	Germany	C	454	Target therapy plus Immunotherapy VS Target therapy	Bevacizumab plus Atezolizumab VS Sorafenib	QoL	EORTC QLQ-C30 and QLQ-HCC18	PO	12.5
G. K. Abou-Alfa (70)	2022	Multiple	America	C	1171	Immunotherapy plus Immunotherapy VS Immunotherapy VS Target therapy	Tislelizumab plus Durvalumab VS Durvalumab VS Sorafenib	QoL	EORTC QLQ-C30	SO	3.5
B. Sangro (71)	2024	Multiple	Spain	C	1171	Immunotherapy plus Immunotherapy VS Immunotherapy VS Target therapy	Tislelizumab plus Durvalumab VS Durvalumab VS Sorafenib	QoL	EORTC-QLQ-C30 and QLQ-HCC18	SO	14.0
B. Sangro (72)	2025	Multiple	Spain	C	616	Target therapy plus Immunotherapy plus Chemotherapy VS Immunotherapy plus placebo VS placebo	durvalumab plus Bevacizumab plus Chemotherapy VS durvalumab plus placebo VS placebo	QoL	EORTC-QLQ-C30 and QLQ-HCC18	SO	4.0
D. Koeberle (73)	2014	Multiple	Switzerland	E	106/88	Target therapy plus Target therapy VS Target therapy	Sorafenib plus Everolimus VS Sorafenib	QoL	FACT-Hep	SO	5.0
C. X. Qiao (74)	2015	Single	China	E	40/33	Target therapy plus traditional Chinese medicine VS Target therapy	Sorafenib plus traditional Chinese medicine VS Sorafenib	QoL	FACT-Hep	SO	5.5
X.L. Chen (75)	2015	Single	China	E	90/50	Target therapy plus Chemotherapy plus radiation therapy VS Target therapy plus Chemotherapy	gefitinib plus Chemotherapy plus radiation therapy VS gefitinib plus Chemotherapy	QoL	EORTC QLQ-C30	PO	9.0
H.M. Liu (76)	2015	Single	China	E	156	Immunotherapy plus Interventional treatment VS Immunotherapy	CIK plus Interventional treatment VS CIK	QoL	EORTC QLQ-C30	PO	7.0
H.X. Zhu (77)	2015	Single	China	E	90/50	Target therapy plus Chemotherapy VS Chemotherapy	gefitinib plus Chemotherapy VS Chemotherapy	QoL	EORTC QLQ-C30	PO	6.0
								Pain	VAS		
C.X. Qiao (78)	2017	Single	China	E	80/73	Target therapy plus Immunotherapy VS Target therapy	CIK plus Sorafenib VS Sorafenib	QoL	FACT-Hep	SO	7.5
S.P. Jiang (79)	2018	Single	China	E	48/27	Target therapy plus Immunotherapy VS Target therapy	Sorafenib plus CIK VS Sorafenib	QoL	FACT-Hep	PO	6.5
J.X. Huang (80)	2021	Single	China	E	62/42	Immunotherapy plus Chemotherapy VS Chemotherapy	Camrelizumab plus Chemotherapy VS Chemotherapy	QoL	QOL-LC	SO	6.0
								Pain	VAS		
P.T. Zhai (81)	2021	Single	China	E	100/52	Target therapy plus Interventional treatment VS Interventional treatment	Apatinib plus Interventional treatment VS Interventional treatment	QoL	SF-36	SO	6.0
X.Y. Wang (82)	2021	Single	China	E	120/81	Targeted combined immunotherapy plus Chemotherapy plus Psychiatric Nursing VS Chemotherapy plus Routine nursing mode	Sorafenib plus Chemotherapy plus Psychiatric Nursing VS Chemotherapy plus Routine nursing mode	QoL	SF-36	SO	6.5
Z.F. Deng (83)	2022	Single	China	E	100/58	Target therapy plus Immunotherapy VS Target therapy plus Immunotherapy	Lenvatinib plus Camrelizumab VS Sorafenib plus Camrelizumab	QoL	SF-36	SO	6.0
W.Q. Li (84)	2022	Single	China	E	100/67	Target therapy plus Immunotherapy VS Target therapy	Lenvatinib plus Sintilimab VS Lenvatinib	QoL	WHOQOL-BREF	SO	6.0
P. Ma (85)	2022	Single	China	E	58/34	Target therapy plus Chemotherapy VS Chemotherapy	Lenvatinib plus Chemotherapy VS Chemotherapy	Functional Assessment of Cancer Therapy	FACT-G	SO	4.5
F.Q. Zhang (86)	2022	Single	China	E	80/63	Target therapy plus Chemotherapy VS Chemotherapy	Lenvatinib plus Chemotherapy VS Chemotherapy	QoL	SF-36	SO	6.0
F. Cao (87)	2023	Single	China	E	40/25	Target therapy plus Immunotherapy plus Chemotherapy VS Chemotherapy	Lenvatinib plus Sorafenib plus Camrelizumab plus Chemotherapy VS Chemotherapy	QoL	SF-36	SO	5.5
L. Di (88)	2023	Single	China	E	104/62	Target therapy plus Immunotherapy VS Target therapy	Lenvatinib plus Toripalimab VS Lenvatinib	QoL	EORTC QLQ-C30	SO	5.0
Y.S. Fu (89)	2023	Single	China	E	102/58	Immunotherapy plus Interventional treatment VS Target therapy	Sintilimab plus Interventional treatment VS Interventional treatment	QoL	SF-36	SO	7.0
Z.C. Hu (90)	2023	Single	China	E	82/48	Target therapy plus Immunotherapy plus Interventional treatment VS Target therapy plus Interventional treatment	Sorafenib plus NK* cell immunotherapy plus Interventional treatment VS Sorafenib plus Interventional treatment	QoL	FACT-G	SO	6.0
Q.H. Liao (91)	2023	Single	China	E	70/39	Target therapy plus Immunotherapy plus Chemotherapy plus Chemotherapy VS Target therapy plus Immunotherapy plus Chemotherapy	Lenvatinib plus Camrelizumab plus Chemotherapy plus Chemotherapy VS Lenvatinib plus Camrelizumab plus Chemotherapy	QoL	EORTC QLQ-C30	SO	6.0
H.H. Ye (92)	2023	Single	China	E	68	Immunotherapy plus Chemotherapy VS Chemotherapy	Tislelizumab plus Chemotherapy VS Chemotherapy	QoL	FACT-G	SO	6.0
Z.H. Liu (93)	2024	Single	China	E	62	Target therapy plus Chemotherapy VS Chemotherapy	Sorafenib plus Chemotherapy VS Chemotherapy	Pain	VAS	SO	4.0
Y. Qu (94)	2024	Single	China	E	90	Immunotherapy plus surgery VS surgery	CIK plus surgery VS surgery	QoL	SF-36	SO	5.0
X.L. Wu (95)	2024	Single	China	E	108	Target therapy plus Immunotherapy plus Chemotherapy VS Target therapy plus Chemotherapy	Toripalimab plus Lenvatinib plus Chemotherapy VS Lenvatinib plus Chemotherapy	QOL	FACT-G	SO	5.0
F. Yang (96)	2024	Single	China	E	92	Immunotherapy plus Immunotherapy plus Chemotherapy VS Immunotherapy plus Chemotherapy	CIK plus Pembrolizumab plus Chemotherapy VS CIK plus Chemotherapy	QoL	EORTC QLQ-C30	SO	5.5
L.F. Yang (97)	2024	Single	China	E	64	Target therapy plus Immunotherapy VS Target therapy	Camrelizumab plus Lenvatinib VS Lenvatinib	QOL	EORTC QLQ-C30	SO	6.0
X. Yin (98)	2024	Single	China	E	102	Target therapy plus Target therapy VS Target therapy	Sorafenib plus Ramucirumab VS Sorafenib	QoL	SF-36	SO	4.5
G.H. Zhong (99)	2024	Single	China	E	58	Target therapy plus Immunotherapy plus Chemotherapy plus Chemotherapy VS Chemotherapy plus Chemotherapy	Bevacizumab plus Sintilimab plus Chemotherapy plus Chemotherapy VS Chemotherapy plus Chemotherapy	Sleep Quality	PSQI	SO	5.0
Y. Chen (100)	2025	Single	China	E	60	Target therapy plus Target therapy VS Target therapy	Sorafenib plus Apatinib VS Sorafenib	QoL	SF-36	SO	7.5
H. Fang (101)	2025	Single	China	E	300	Target therapy plus Chemotherapy VS Target therapy	Sorafenib plus Chemotherapy VS Sorafenib	QoL	SF-36	SO	5.0
J.Y. Yan (102)	2025	Single	China	E	112	Immunotherapy plus Interventional treatment VS Interventional treatment	Atezolizumab plus Interventional treatment VS Interventional treatment	QoL	EORTC QLQ-C30	SO	5.0
D.Y. Zhu (103)	2025	Single	China	E	62	Immunotherapy plus Chemotherapy VS Chemotherapy	Camrelizumab plus Chemotherapy VS Chemotherapy	QoL	QLACS	SO	4.5
Z. Ren (104)	2015	Multiple	China	O	871/745	Target therapy plus BSC* VS Target therapy	Sorafenib plus BSC VS Sorafenib	Hand-foot skin reaction and QoL	HF-QoL	SO	6.5
J. Guo (105)	2016	Single	China	O	60/31	Immunotherapy plus Optimize nursing intervention VS Immunotherapy plus Routine nursing mode	CIK plus Optimize nursing intervention VS CIK plus Routine nursing mode	Anxiety	SAS	PO	7.5
								Depression	SDS		
H.M. Liu (106)	2016	Single	China	O	156/95	Immunotherapy plus cognitive intervention VS Immunotherapy plus Routine nursing mode	CIK plus Cognitive intervention VS CIK plus Routine nursing mode	QoL	EORTC QLQ-C30	PO	9.0
								Pain	VAS		
J. Kuang (107)	2020	Single	China	O	96/58	Target therapy plus surgery plus ERAS* VS Target therapy plus surgery plus Routine nursing mode	Sorafenib plus surgery plus ERAS* VS Sorafenib plus surgery plus Routine nursing mode	QoL	EORTC QLQ-C30	SO	3.0
J.Z. Chen (108)	2021	Single	China	O	58/31	Target therapy plus High quality nursing VS Target therapy plus Routine nursing mode	No reported	QoL	GQOLI-74	SO	6.5
								Sleep Quality	PSQI		
Y.J. Deng (109)	2021	Single	China	O	66/47	Target therapy plus traditional Chinese medicine VS Target therapy	Sorafenib plus traditional Chinese medicine VS Sorafenib	QoL	QOL-LC	PO	5.5
S. Wang (110)	2021	Single	China	O	100/54	Target therapy plus humanistic nursing plus Narrative Nursing VS Target therapy plus humanistic nursing	Sorafenib plus humanistic nursing plus Narrative Nursing plus VS Sorafenib plus humanistic nursing	Well-being	Campbell IWB	PO	5.0
								Negative emotions	MCMQ		
								QoL	EORTC QLQ-C30		
Y.L. Yang (111)	2022	Single	China	O	140/90	Target therapy plus CNP* VS Target therapy plus Routine nursing mode	Sorafenib plus CNP VS Sorafenib plus Routine nursing mode	Sleep Quality	PSQI	PO	5.5
								QoL	EORTC QLQ-C30		
Y.F. Zhang (112)	2022	Single	China	O	88/43	Target therapy plus Chemotherapy VS Target therapy	Sorafenib plus Chemotherapy VS Sorafenib	Sleep Quality	PSQI	PO	5.0
								Anxiety	SAS		
								Depression	SDS		
Y. Liu (113)	2023	Single	China	O	120/82	Target therapy plus Chemotherapy plus Orem’s Self-Care Model VS Target therapy plus Chemotherapy plus Routine nursing mode	Sorafenib plus Chemotherapy plus Orem’s Self-Care Model VS Sorafenib plus Chemotherapy plus Routine nursing mode	SCA	ESCA	PO	7.0
								Anxiety	SAS		
								Depression	SDS		
L.Q. Yao (114)	2023	Single	China	O	80/51	Target therapy plus Immunotherapy plus Exercise - Psychological - Sleep Nursing Intervention plus Aromatherapy VS Target therapy plus Immunotherapy plus Routine nursing mode	No reported	Sleep Quality	PSQI and DBAS	PO	8.5
								QoL	EORTC QLQ-C30		
								Patient satisfaction	Self-made patient satisfaction survey form		
D. Yu (115)	2023	Single	China	O	112/69	Immunotherapy plus traditional Chinese medicine VS Immunotherapy	Nivolumab/Sintilimab/Camrelizumab plus traditional Chinese medicine VS Nivolumab/Sintilimab/Camrelizumab	Pain	NRS	SO	5.5
Y.H. Zhu (116)	2023	Single	China	O	70/53	Immunotherapy plus traditional Chinese medicine VS Immunotherapy	No reported	Dermatology Life Quality	DLQI	SO	5.5
L.J. Deng (117)	2024	Single	China	O	60	Target therapy plus Cloud hospital nursing management model VS Target therapy plus Routine nursing mode	No reported	Medication Adherence	MMAS-8	SO	6.5
								Managerial self-efficacy	SUPPH		
								SCA	ESCA		
D.Y. Jiang (118)	2024	Single	China	O	80	Immunotherapy plus High quality nursing VS Immunotherapy plus Routine nursing mode	No reported	Anxiety	SAS	PO	7.0
								Depression	SDS		
								Cognitive function	MMSE		
								Sleep Quality	PSQI		
								QoL	SF-36		
P. Lu (119)	2024	Single	China	O	120	Target therapy plus Psychological intervention based on dignity therapy VS Target therapy plus Routine nursing mode	No reported	Medication Adherence	MMAS-8	PO	7.0
								Patient Dignity	PDI		
								Self-Perceived Burden	SPBS		
								QoL	EORTC QLQ-C30		
P.Y. Yang (120)	2024	Single	China	O	60	Target therapy plus traditional Chinese medicine plus traditional Chinese medicine VS Target therapy plus traditional Chinese medicine	Sorafenib plus traditional Chinese medicine plus traditional Chinese medicine VS Sorafenib plus traditional Chinese medicine	QoL	SF-36	SO	4.5
Y. Yao (121)	2024	Single	China	O	93	Target therapy plus traditional Chinese medicine VS Target therapy	Lenvatinib plus traditional Chinese medicine VS Lenvatinib	QoL	EORTC QLQ-HCC18	SO	4.0
K. Zhou (122)	2024	Single	China	O	61	Target therapy plus Immunotherapy plus traditional Chinese medicine VS Target therapy plus Immunotherapy	Bevacizumab plus Tislelizumab plus traditional Chinese medicine VS Bevacizumab plus Tislelizumab	QoL	EORTC QLQ-C30	SO	5.5
								Pain	VAS		

Explanations:.

PROs, Patient-Reported Outcomes; PROMs, Patient-Reported Outcomes Measures; SO, Secondary Outcome; PO, Primary Outcome; CONSORT, Consolidated Standards of Reporting Trials; BSC, Best Supportive Care; ERAS, Enhanced Recovery After Surgery; CNP, Clinical Cursing Pathways; CIK, Cytokine-Induced Killer; NK, Natural Killer; QoL, Quality of Life; HRQoL, Health-Related Quality of Life; SCA, Self-Care Agency; FACT-Hep, Functional Assessment of Cancer Therapy-Hepatobiliary; HF-QoL, Hand-Foot Skin Reaction and Quality of Life Questionnaire; EORTC QLQ-C30, European Organization for Research and Treatment of Cancer Quality of Life Questionnaire; VAS, Visual Analogue Scale; EQ-5D, EuroQol Five Dimensions Questionnaire; QLQ-HCC18, Quality of Life Questionnaire-Hepatocellular Carcinoma 18; SAS, Self-Rating Anxiety Scale; SDS, Self-rating depression scale; GQOLI-74, Generic Quality of Life Inventory-74; PSQI, Pittsburgh sleep quality index; QoL-LC, Quality Of Life-Liver; SF-36, Short Form-36 Health Survey; IWB, index of well-being; MCMQ, Medical Coping Modes Questionnaire; WHOQOL-BREF, World Health Organization Quality of Life - Brief Version; FACT-G, Functional Assessment of Cancer Therapy-General; ESCA, Self-Care Ability Scale; DBAS, Dysfunctional Beliefs and Attitudes on Sleep; NRS, Numerical Rating Scale; DLQI, Dermatology Life Quality Index; MMAS-8, Morisky Medication Adherence Scale-8; SUPPH, strategies used by people to promote health; MMSE, Minimum Mental State Examination; PDI, Patient Dignity Inventory; SPBS, Self-Perceived Burden Scale; QLACS, Quality of Life in Adult Cancer Survivors.

Study Type: C, Confirmatory: Phase III trials or large-scale registrational trials designed to support licensing or establish definitive efficacy. E, Exploratory: Phase I/II trials or smaller-scale efficacy studies designed to explore preliminary efficacy or estimate dosing. O, Other: Nursing interventions, traditional Chinese medicine studies, supportive care evaluations, and mechanism-focused studies that do not conform to the traditional phase classification.

Sixty-four trials involved 8,494 participants, ranging from 40 to 1171 with an average of 133 per trial. 74.58% participants were male. Of the included trials, 62 were double-arm trials and 2 were triple-arm trials. In the observation group of the double-arm trials, 7 studies applied single therapy and 55 studies applied combination therapy. In the control arm, 40 studies adopted single therapy and 22 employed combination therapy. The combination regimens included the combinations of different drugs within the same treatment modality. It also involved combinations across different treatment modalities, and the integration of the aforementioned combination regimens with supportive care. Among 50 trials focusing on targeted therapy, sorafenib was used the most frequently, followed by lenvatinib and bevacizumab. Immunotherapy was employed in 32 trials, with Cytokine-Induced Killer (CIK) cell therapy being the most frequently used, followed by sintilimab and atezolizumab. Only 15 studies (15/64) designated PROs as the primary outcome, while the majority (49/64) utilized PROs as the secondary outcome (see **Table 1**).

### 3.3 The application of PROs and PROMs

Of the included studies, 27 PROMs referring to 17 relevant domains of PRO were observed. Eight symptomatic adverse events (sleep quality, pain, anxiety, depression, dermatology, hand-foot skin reaction, negative emotions and cognitive function) were reported. Ten PRO reports in the nursing field documented 16 PROMs covering 12 domains and 54 reports in the medical field employed 17 PROMs addressing 10 domains. The core domain of PRO primarily included the broad QoL (54/64), as well as single-domain symptoms or specific functioning (19/64). The broad PROMs for QoL are categorized into generic and specific measures. The frequently used generic assessment instruments for QoL were the European Organization for Research and Treatment of Cancer Quality of Life Questionnaire (EORTC QLQ-C30) (25/64) and the Short Form-36 Health Survey (SF-36) (12/64). The EORTC QLQ-C30 is a cancer-specific measure covering the domains of functional, symptom burden, and global health (27). The SF-36 is a generic measure with eight health dimensions, including physical functioning and social role functioning (28) (see **Tables 1**, **2**).

**Table 2:** The characteristics of patient-reported outcome in the included studies (*N* = 64).

PROs	PROMs	*N*(%)	PROs	PROMs	*N*(%)
QoL	EORTC QLQ-C30	17(26.6)	Sleep Quality	PSQI	5(7.8)
	SF-36	12(18.8)		PSQI、DBAS	1(1.6)
	FACT-Hep	4(6.3)	Pain	VAS	5(7.8)
	FACT-G	3(4.7)		NRS	1(1.6)
	QoL-LC	2(3.1)	Anxiety	SAS	4(6.3)
	EORTC QLQ-HCC18	1(1.6)	Depression	SDS	4(6.3)
	WHOQOL-BREF	1(1.6)	SCA	ESCA	2(3.1)
	QLACS	1(1.6)	Dermatology life quality	DLQI	1(1.6)
	GQOLI-74	1(1.6)	Hand-foot skin reaction and QoL	HF-QoL	1(1.6)
	EORTC QLQ-C30 plus QLQ-HCC18	6(9.4)	Managerial self-efficacy	SUPPH	1(1.6)
	EORTC QLQ-C30 plus QLQ-HCC18 plus EQ-5D	1(1.6)	Well-being	Campbell IWB	1(1.6)
	EORTC QLQ-C30 plus FACT-Hep	1(1.6)	Patient dignity	PDI	1(1.6)
	FACT-Hep plus EQ-5D	1(1.6)	Functional assessment of cancer therapy	FACT-G	1(1.6)
HRQoL	EQ-5D	1(1.6)	Medication adherence	MMAS-8	1(1.6)
	EQ-5D plus EQ-VAS	1(1.6)	Negative emotions	MCMQ	1(1.6)
Physical wellbeing	FACT-Hep	1(1.6)	Cognitive function	MMSE	1(1.6)

PROs, Patient-Reported Outcomes; PROMs, Patient-Reported Outcomes Measures; QoL, Quality of Life; HRQoL, Health-Related Quality of Life; SCA, Self-Care Agency; EORTC QLQ-C30, European Organization for Research and Treatment of Cancer Quality of Life Questionnaire; SF-36, Short Form-36 Health Survey; FACT-Hep, Functional Assessment of Cancer Therapy-Hepatobiliary; FACT-G, Functional Assessment of Cancer Therapy-General; QoL-LC, Quality Of Life-Liver; EORTC QLQ-HCC18, European Organization for Research and Treatment of Cancer Quality of Life Questionnaire-Hepatocellular Carcinoma 18; WHOQOL-BREF, World Health Organization Quality of Life-Brief Version; QLACS, Quality of Life in Adult Cancer Survivors; GQOLI-74, Generic Quality of Life Inventory-74; EQ-5D, EuroQol Five Dimensions Questionnaire; EQ-VAS, EuroQol Visual Analogue Scale; PSQI, Pittsburgh sleep quality index; DBAS, Dysfunctional Beliefs and Attitudes on Sleep; VAS, Visual Analogue Scale; NRS, Numerical Rating Scale; SAS, Self-Rating Anxiety Scale; SDS, Self-rating depression scale; ESCA, Self-Care Ability Scale; DBAS, Dysfunctional Beliefs and Attitudes on Sleep; DLQI, Dermatology Life Quality Index; HF-QoL, Hand-Foot Skin Reaction and Quality of Life Questionnaire; SUPPH, strategies used by people to promote health; IWB, index of well-being; PDI, Patient Dignity Inventory; MMAS-8, Morisky Medication Adherence Scale-8; MCMQ, Medical Coping Modes Questionnaire; MMSE, Minimum Mental State Examination.

The frequently employed disease-specific instruments for QoL were the European Organization for Research and Treatment of Cancer Quality of Life Questionnaire-Hepatocellular Carcinoma 18 (EORTC QLQ-HCC18) (8/64) and the Functional Assessment of Cancer Therapy-Hepatobiliary (FACT-Hep) (7/64). The EORTC QLQ-HCC18 is a supplementary module to the EORTC QLQ-C30, specifically designed for assessing QoL in liver cancer patients. The FACT-Hep is a disease-specific module of the Functional Assessment of Cancer Therapy-General (FACT-G) scale designed to assess the specific QoL in liver cancer patients. They are frequently used with generic measures like the QLQ-C30 or EuroQol Five Dimensions Questionnaire (EQ-5D) (29). Besides, the Quality-of-Life Questionnaire for Liver Cancer (QOL-LC) (30) has been primarily used in studies conducted in Asia. In particular, the Dermatology Life Quality Index (DLQI) (31) was applied to assess skin reaction-related QoL in liver cancer patients, while the Hand-Foot Quality of Life questionnaire (HF-QoL) (32) was used to evaluate the impact of drug-induced hand-foot skin reactions on QoL.

The frequently utilized instruments for assessing single-domain symptoms or specific functioning were the Pittsburgh Sleep Quality Index (PSQI) (34) (6/64), the Visual Analogue Scale (VAS) (35) (5/64), the Self-Rating Anxiety Scale (SAS) (36) (4/64), and the Self-Rating Depression Scale (SDS) (37) (4/64). The PSQI was used to assess sleep quality, the VAS for pain, the SAS for anxiety, and the SDS for depression. In nursing research, the Self-Care Ability Evaluation Scale (ESCA) (33) was frequently used to assess patients’ self-care ability (2/64).

**Figure 2:**
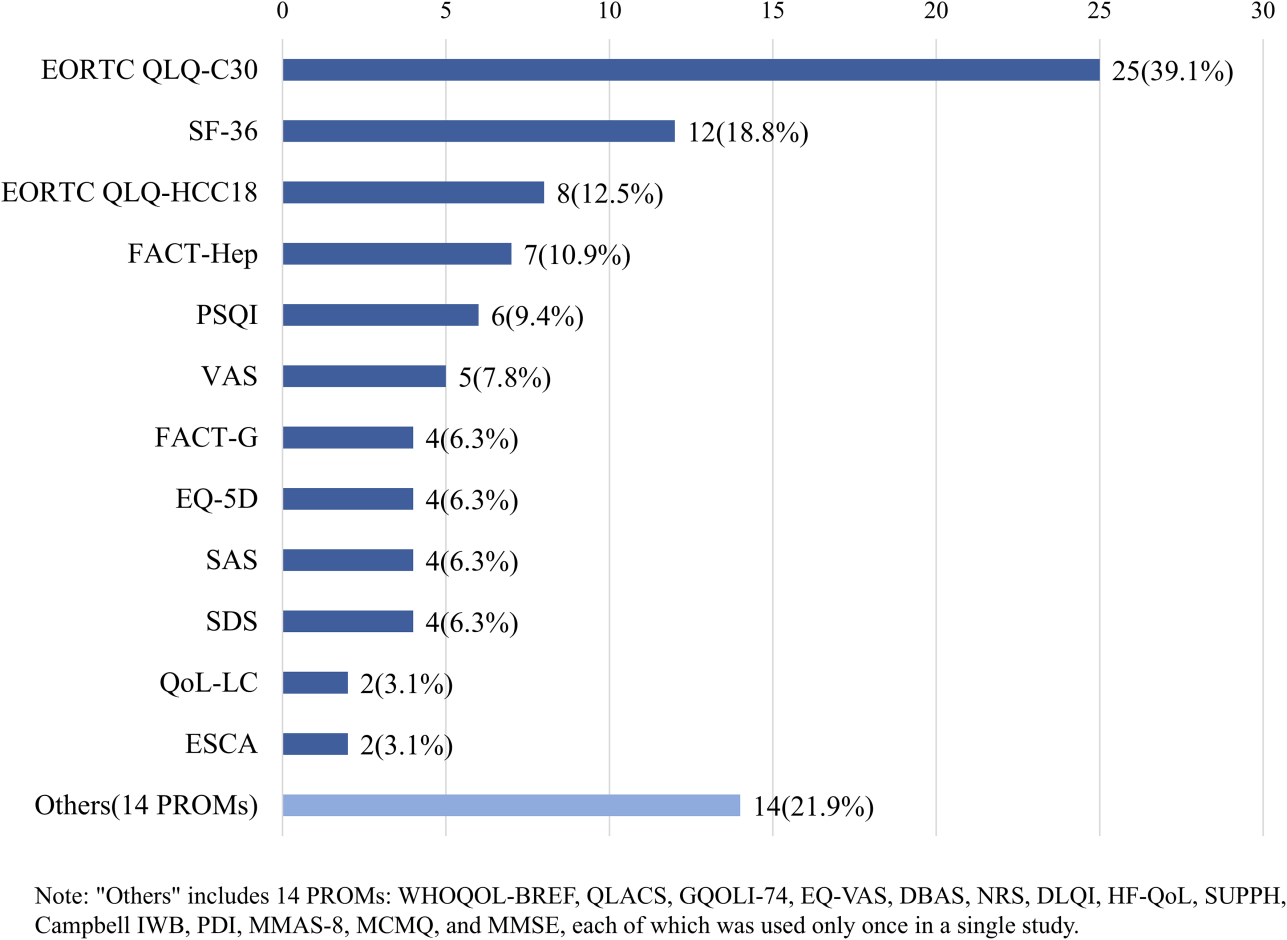
Frequency of PROMs used in included studies (N = 64).

Among the 64 included studies, the most frequently used PROM was the EORTC QLQ-C30 which was employed in 25 studies (39.1%), followed by the SF-36 which was used in 12 studies (18.8%). The disease-specific instruments of the EORTC QLQ-HCC18 was applied in 8 studies (12.5%), and the FACT-Hep was utilized in 7 studies (10.9%) (see **Table 2**, **Figure 2**). The most frequently used PROM was the EORTC QLQ-C30 (15/50) in Chinese studies, followed by the SF-36 (12/50). The most commonly used PROMs instrument was also the EORTC QLQ-C30 (10/14), followed by the EORTC HCC-18 (7/14) in non-Chinese studies (see **Table 1**). Last but not least, 40 studies employed a single PROM, while 24 studies utilized multiple PROMs. Among studies using multiple PROMs, the predominant combination patterns were the use of a generic QoL instrument alongside a single-domain PROM (9/24) and the combination of a generic with a disease-specific QoL instrument (10/24) (see **Table 1**).

### 3.4 The reporting quality of PRO

The reporting quality of PROs in the included studies was assessed based on the CONSORT 2010 and the CONSORT-PRO (see **Table 3**). The results showed that only 7.8% of studies clearly defined PROs as primary or secondary outcomes in their abstracts, 92.2% did not describe the methods for handling missing PRO data and 90.6% did not report PRO at the domain level. Within the CONSORT-PRO items, reporting adherence was good in 4 items (Item 13ai, Item 13aii, Item 16, Item 22), moderate in 4 (Item P1b partially addressed, Item P6ai, Item 15, Item 17ai), and poor in another 12 items. In contrast, items P7a (how sample size was determined) and P12a (statistical approach for dealing with missing data) were the most often neglected items in the reports.

**Table 3:** The reporting quality of PRO in the included studies according to the CONSORT 2010 and CONSORT-PRO statement (*N* = 64).

CONSORT 2010 and CONSORT-PRO item^a^		Complete report	Not complete report	No report
		*N*(%)	*N*(%)	*N*(%)
1a	Identification as a randomized trial	10(15.6)	32(50.0)	22(34.4)
1b	Structured summary of the trial design, methods, results, and conclusions	60(93.8)	2(3.1)	2(3.1)
**P1b**	**The PRO should be identified in the abstract as a primary or secondary outcome**	5(7.8)	49(76.6)	10(15.6)
**2a**	**Scientific background and explanation of rationale**	12(18.8)	51(79.7)	1(1.6)
2b	Specific objectives or hypotheses	55(85.9)	8(12.5)	1(1.6)
**P2b**	**The PRO hypothesis should be stated and relevant domains identified, if applicable**	6(9.4)	33(51.6)	25(39.1)
3a	Description of trial design (such as parallel, factorial) including allocation ratio	62(96.9)	1(1.6)	1(1.6)
3b	Important changes to methods after trial commencement (such as eligibility criteria), with reasons	3(4.7)	0	61(95.3)
4a	Eligibility criteria for participants	64(100.0)	0	0
4b	Settings and locations where the data were collected	56(87.5)	5(7.8)	3(4.7)
5	The interventions for each group with sufficient details to allow replication, including how and when they were actually administered	21(32.8)	42(65.6)	1(1.6)
6a	Completely defined prespecified primary and secondary outcome measures, including how and when they were assessed	31(48.4)	33(51.6)	0
**P6a**	**Evidence of PRO instrument validity and reliability should be provided or cited if available, including the person completing the PRO and methods of data collection (paper telephone electronic other)**	6(9.4)	48(75.0)	10(15.6)
6b	Any changes to trial outcomes after the trial commenced, with reasons	2(3.1)	0	62(96.9)
**7a**	**How sample size was determined**	14(21.9)	1(1.6)	49(76.6)
7b	When applicable, explanation of any interim analyses and stopping guidelines	8(12.5)	2(3.1)	54(84.4)
8a	Method used to generate the random allocation sequence	44(68.8)	3(4.7)	17(26.6)
8b	Type of randomization; details of any restriction (such as blocking and block size)	10(15.6)	6(9.4)	48(75.0)
9	Mechanism used to implement the random allocation sequence (such as sequentially numbered containers), describing any steps taken to conceal the sequence until interventions were assigned	9(14.1)	3(4.7)	52(81.3)
10	Who generated the random allocation sequence, who enrolled participants, and who assigned participants to interventions	10(15.6)	1(1.6)	53(82.8)
11a	If done, who was blinded after assignment to interventions (for example, participants, care providers, those assessing outcomes) and how	7(10.9)	1(1.6)	56(87.5)
11b	If relevant, description of the similarity of interventions	16(25.0)	41(64.1)	7(10.9)
12a	Statistical methods used to compare groups for primary and secondary outcomes	13(20.3)	50(78.1)	1(1.6)
12b	Methods for additional analyses, such as subgroup analyses and adjusted analyses	11(17.2)	0	53(82.8)
**P12a**	**Statistical approaches for dealing with missing data are explicitly stated**	5(7.8)	0	59(92.2)
**13a**	**For each group, the numbers of participants who were randomly assigned, received intended treatment, and were analyzed for the primary outcome**	55(85.9)	8(12.5)	1(1.6)
13b	For each group, losses and exclusions after randomization, together with reasons	13(20.3)	0	51(79.7)
14a	Dates defining the periods of recruitment and follow-up	10(15.6)	13(20.3)	41(64.1)
14b	Why the trial ended or was stopped	6(9.4)	0	58(90.6)
**15**	**A table showing baseline demographic and clinical characteristics for each group**	7(10.9)	40(62.5)	17(26.6)
**16**	**For each group, number of participants (denominator) included in each analysis and whether the analysis was by original assigned groups**	59(92.2)	0	5(7.8)
**17a**	**For each primary and secondary outcome, results for each group, and the estimated effect size and its precision (such as 95% confidence interval)**	9(14.1)	54(84.4)	1(1.6)
17b	For binary outcomes, presentation of both absolute and relative effect sizes is recommended	9(14.1)	10(15.6)	45(70.3)
**18**	**Results of any other analyses performed, including subgroup analyses and adjusted analyses, distinguishing pre-specified from exploratory**	13(20.3)	0	51(79.7)
19	All important harms or unintended effects in each group	35(54.7)	4(6.3)	25(39.1)
20	Trial limitations, addressing sources of potential bias, imprecision, and, if relevant, multiplicity of analyses	21(32.8)	4(6.3)	39(60.9)
21	Generalizability (external validity, applicability) of the trial findings	63(98.4)	0	1(1.6)
**22**	**Interpretation consistent with results, balancing benefits and harms, and considering other relevant evidence**	55(85.9)	8(12.5)	1(1.6)
**P20/21**	**PRO-specific limitations and implications for generalizability and clinical practice should be discussed**	6(9.4)	16(25.0)	42(65.6)
23	Registration number and name of trial registry	14(21.9)	1(1.6)	49(76.6)
24	Where the full trial protocol can be accessed, if available	12(18.8)	1(1.6)	51(79.7)
25	Sources of funding and other support (such as supply of drugs), role of funders	16(25.0)	0	48(75.0)

PRO, patient-reported outcome; CONSORT, Consolidated Standards of Reporting Trials.

^a^ CONSORT PRO items are highlighted in bold.

**Figure 3:**
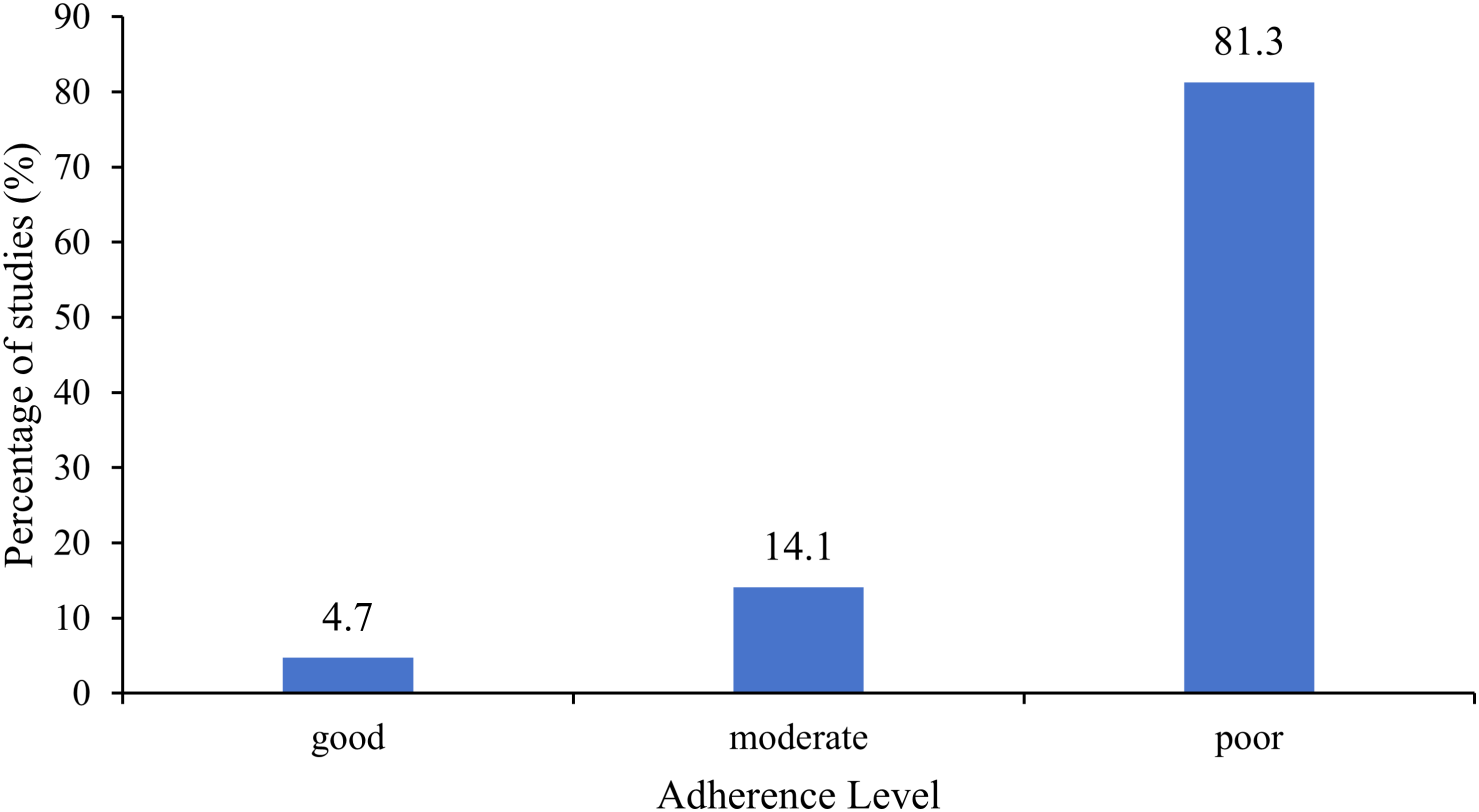
Distribution of studies’ CONSORT-PRO adherence levels (N = 64).

The mean score of the CONSORT-PRO adherence was 6.2 (ranging from 3 to 14) in all included studies. Most studies did not report or incompletely reported the items (see **Table 4**). Besides, the mean score of the CONSORT-PRO adherence was 7.5 (ranging from 3 to 14) for confirmatory studies, 5.8 (ranging from 4 to 9) for exploratory studies, and 6.0 (ranging from 3 to 9) for other studies. Confirmatory studies demonstrated higher CONSORT-PRO adherence scores than both exploratory and other studies. Notable variability in the quality of PRO reporting was observed among confirmatory studies (scores ranging from 3 to 14), of which 28.6% had scores of ≥10 and 42.9% had scores of ≤5. The mean score of the CONSORT-PRO adherence was 5.9 (ranging from 4 to 9) for studies conducted in China and 7.4 (ranging from 3 to 14) for non-Chinese studies (see **Table 3**). The Chinese studies showed lower CONSORT-PRO adherence scores than other non-Chinese studies. Of the 64 included studies, only 3 studies demonstrated a good level of CONSORT-PRO adherence, 9 exhibited a moderate level of compliance, and the remaining 52 showed a poor level of CONSORT-PRO adherence (see **Figure 3**).

**Table 4:** CONSORT-PRO item adherence scores in the included studies (*N* = 64).

CONSORT-PRO item	RCT addressing the item (*N* = 64)		
	*N*	%	Compliance rating
P1b. Abstract—PRO as primary/secondary endpoint			
Item P1b completely addressed	5	7.8	poor
Item P1b partially addressed	49	76.6	Moderate
2a. Rationale for including PRO endpoint	24	37.5	poor
P2bi. PRO hypothesis present	11	17.2	poor
P2bii. PRO domains in hypothesis	6	9.4	poor
P6ai. Evidence of PRO instrument validity	33	51.6	Moderate
P6aii. Statement of the person completing the PRO questionnaire	15	23.4	poor
P6aiii. Mode of administration (paper, e-PRO)	6	9.4	poor
P7a. How sample size was determined (not required unless PRO is a primary endpoint)	0	0.0	poor
P12a. Statistical approach for dealing with missing data (imputation, exclusion, other)	5	7.8	poor
13ai. Report no. questionnaires submitted/available for analysis at baseline	57	89.1	good
13aii. Report no. questionnaires submitted/available for analysis principle time point for analysis	55	85.9	good
15. Demographics table includes baseline PRO	47	73.4	Moderate
16. Number of pts (denominator) included in each PRO analysis	59	92.2	good
17ai. PRO results reported for the hypothesized domains and time point specified in the hypothesis-OR-reported for each domain of the PRO questionnaire if no PRO hypothesis provided	50	78.1	Moderate
17aii. Results include confidence interval, effect size or some other estimate of precision	15	23.4	poor
18. Results of any subgroup/adjusted/exploratory analyses	13	20.3	poor
P20. PRO study limitations	6	9.4	poor
P21. Implications of PRO results for generalizability, clinical practice	16	25.0	poor
22. PROs interpreted in relation to clinical outcomes	55	85.9	good

CONSORT, Consolidated Standards of Reporting Trials; PRO, Patient-Reported Outcome; RCT, randomized controlled trail; N/A, not applicable.

Compliance rating cut-off scores: “good” = >80% of RCTs within the group addressed the item; “moderate” = 50–79% of RCTs within the group addressed the item; “poor” = ≤49% RCTs within the group addressed the item.

## 4 Discussion

This scoping review included 64 RCTs focusing on targeted therapy and/or immunotherapy for liver cancer. The publication time of the included literature demonstrated a phased increase. Except for one study published in 2009, 18 studies (28.1%) were published between 2014 and 2020, and 45 studies (70.3%) were published from 2021 to 2025. Over the last five years, the use of PROs in RCTs of targeted therapy and/or immunotherapy for liver cancer has markedly risen. This trend may be related to the key technological breakthroughs and changes in systemic treatment for liver cancer (4, 7, 34). In addition, the publication of the 2013 CONSORT-PRO guideline provided a framework for standardizing the use of PROs in research (19, 35), which has heightened researchers’ attention to PROs. Notably, the majority of studies (78.1%) were conducted in China, which might be associated with the substantial population of liver cancer patients in China and prevailing policy preferences (36, 37). Besides, this geographical distribution may also be related to our search strategy, which covered both international and Chinese databases and was restricted to English and Chinese publications.

Among the 64 studies, only 15 (23.4%) defined PROs as the primary outcome, while 49 (76.6%) classified PROs as a secondary outcome. This result indicated that PROs were often utilized as supplementary outcomes in current RCTs of targeted therapy and/or immunotherapy. This research design may underestimate the value of PROs in assessing treatment tolerability and patient experience (38). Previous research has shown that while combining targeted therapy and immunotherapy can prolong survival, it may also intensify symptom burden (14). PRO data are essential for balancing treatment efficacy against toxicity and optimizing therapeutic outcomes. Furthermore, the PRO data also has its own independent prognostic value (39). Some researchers argued that the PROs at baseline or during the early stage of treatment were significantly associated with patients’ survival period (40, 41). Therefore, it is crucial to attach the value of PROs in research within the patient-centered care environment.

The included studies covered 27 PROMs about 17 relevant domains. QoL was the most frequently evaluated domain. The EORTC QLQ-C30, a generic tool designed for a broad cancer population, was the most often used PROM for assessing QoL in liver cancer patients. However, this tool fails to comprehensively capture liver cancer-specific symptoms, such as ascites, hepatic pain, jaundice, abdominal distension, etc. The identification of liver cancer-specific symptoms is primarily dependent on the QLQ-HCC18, the FACT-Hep, or other single-domain tools (42). However, only 8 studies concurrently employed both the EORTC QLQ-C30 and the QLQ-HCC18 or the FACT-G and the FACT-Hep. The use of single-domain tools was also limited. Furthermore, previous studies have reported that liver cancer patients receiving targeted therapy and/or immunotherapy often experienced multiple adverse symptoms (43, 44). So, we considered that the assessment of PROs in the included studies might be insufficient to fully capture the symptom burden in this patient population. While using multiple PROMs often increases the reported burden of patients (45). Recent research has shown that computerized adaptive testing (CAT) can alleviate the response burden of patients and improve the quality of PRO data (46). So, the application of CAT for the electronic and personalized management of PRO data is crucial in future research directions. In particular, the exclusive use of generic instruments in confirmatory studies is prone to missing disease-specific symptom information. Therefore, we do not recommend using generic tools as the primary measurement tools for collecting PRO data. An ideal PROM should simultaneously assess both disease-specific symptoms and overall health-related quality of life, while also possessing sound psychometric properties. Thus, we suggest prioritizing the use of disease-specific assessment tools; specifically, the EORTC QLQ-C30 should be used in conjunction with the QLQ-HCC18 module to address the limitations of generic tools in specific disease assessment.

Prior studies have shown that adverse events significantly influence patients’ QoL (47, 48). Basch et al. further emphasized the importance of QoL and proposed that more attention should be paid to dynamically tracking the symptomatic outcomes (49). However, there were relatively few evaluations of symptoms, such as sleep, pain, and hand-foot skin reactions, in the included studies. The scarcity in symptomatic outcomes assessment can be partly attributed to the existing healthcare model. In clinical practice, the reporting of these symptomatic adverse events primarily relies on physicians according to the Common Terminology Criteria for Adverse Events (CTCAE) criteria. However, the accurate capture of common symptomatic adverse events depends on the direct reporting of patients. Yip’s research (50) has also shown that there are significant differences between the symptoms reported by patients and those recorded by medical staff. Physician-driven reporting of symptomatic adverse events often underestimates the incidence and severity of such events (51). The symptoms self-reported by patients are usually earlier, more frequent and more severe. Moreover, existing studies have found that liver cancer patients receiving targeted therapy and/or immunotherapy often experienced multiple adverse symptoms (43, 44). However, most researchers have only employed general tools to assess the overall QoL, which may overlook the disease-specific symptoms and treatment-specific symptoms in those receiving targeted therapy and/or immunotherapy. Al-Naesan (52) noted that generic instruments of QoL often lacked sufficient sensitivity to capture such specific symptoms. The lack of standardized and validated tools for different treatment modalities or diseases, or the inadequate use of such tools, may limit the overall quality of assessment. Therefore, future research should prioritize the development and validation of PRO tools for liver cancer patients who received targeted therapy and/or immunotherapy. These tools are critical to sensitively detect treatment-related adverse events and provide a reliable evidence base for clinical management.

Notably, the studies focusing on nursing intervention assessed a wider range of PROs, including anxiety, depression, and sleep quality and QoL, self-care ability, medication adherence, and overall well-being. In these studies, the purpose of assessing PROs was to evaluate the efficiency of non-pharmacological interventions, and then to improve patients’ experience and daily functioning. However, PROs in nursing research often failed to establish robust associations with clinical endpoints such as overall survival or tumor response rate. Furthermore, the integration of PROs with objective clinical data, including laboratory parameters and imaging findings, remained insufficient. Consequently, the value of nursing interventions may be difficult to quantify and fully acknowledged by clinicians and policymakers, and the translation of PRO findings into specific clinical nursing guidelines becomes complex. In medical research, PROs were primarily used to assess treatment efficacy and safety or to facilitate new drug registration. Whereas the potential of these PRO results to guide the development of personalized clinical care management strategies has not been fully exploited. Therefore, we advocate for the integration of PRO assessments led by nursing experts into large-scale medical RCTs, and to develop a collaborative framework that encompasses medical treatment evaluation, nursing symptom management, and patient experience (53). Future PRO research should adopt a multidisciplinary collaborative approach from the design stage, thereby strengthening the application of PRO evidence in clinical practice for patients with liver cancer.

PROs are essential for obtaining patients’ information that clinicians may not consistently monitor. However, our study revealed that the mean CONSORT-PRO adherence score of the included studies was only 6.2 (3–14). The overall adherence to the CONSORT-PRO checklist was poor in included RCTs on targeted therapy and/or immunotherapy for liver cancer. Similar findings were observed in RCTs for systemic treatment of late-stage soft tissue sarcomas in adults (20), locally recurrent rectal cancer (54), and multiple myeloma (55). What’s more, Kyte’s study indicated that 38% to 80% of trial protocols inadequately addressed or did not report PRO according to the CONSORT-PRO extension (56). This means that among the studies that reported PROs, low-quality PRO reporting may underestimate the value of PROs. Incomplete reporting may mask a potential risk of treatment, compromising the potential benefits for patients, clinicians, and healthcare institutions. Therefore, we recommend that future studies strictly adhere to the CONSORT-PRO guidelines for reporting PROs and cite it in their papers (57).

In addition, our study found that the mean CONSORT-PRO adherence score was higher for confirmatory studies (7.5) than for exploratory studies (5.8) and other studies (6.1). Confirmatory studies are typically intended to support drug marketing authorization and thus need to provide adequate benefit-risk evaluation evidence for regulatory agencies. Moreover, in recent years, regulatory institutes such as the FDA have explicitly required sponsors to include PRO data in drug approvals (58). Such regulatory pressure has prompted trial investigators to collect, analyze, and report PRO data more rigorously, thereby improving the CONSORT-PRO adherence of confirmatory studies. From a trial design perspective, confirmatory studies usually have larger sample sizes, longer follow-up periods, and more robust designs, with greater resources to implement PRO assessments. Finally, according to the ICH E8(R1) guideline, confirmatory studies need to prospectively focus on quality across all research aspects from the protocol design stage, including PRO-related hypotheses, instrument selection, data collection, and handling of missing data. This prospective design planning gives confirmatory trials an advantage in the completeness of PRO reporting. However, we observed significant variability in the quality of PRO reporting among confirmatory studies. The mean score of CONSORT-PRO adherence was ≥10 in 28.6% of confirmatory trials and ≤5 in 42.9% of confirmatory trials. The primary issues contributing to low-quality PRO reporting included failure to report methods for handling missing data, lack of pre-specified PRO hypotheses, and failure to present domain-level results in PRO data. Therefore, there remains a need to continue promoting the CONSORT-PRO guidelines and enforcing the regulatory in confirmatory studies.

In contrast, exploratory studies (such as phase I/II or small-sample efficacy studies) primarily aim to assess the preliminary efficacy of targeted therapy and/or immunotherapy, determine drug dosages, and evaluate safety. In these studies, PROs are often predefined as secondary or exploratory endpoints rather than core primary outcomes. Consequently, researchers invest relatively limited attention and resources in PRO collection and reporting. This research objectives directly lower the quality of PRO reporting.

Studies classified as “Other” (nursing intervention studies, traditional Chinese medicine studies, and supportive care studies, et al.) often fall outside the regulatory framework for drug approval. Researchers in these fields have limited awareness and application of PRO reporting guidelines, which results in poor reporting quality. Notably, in nursing intervention studies and supportive care studies, PROs are frequently used as primary outcome measures. Therefore, improving the quality of PRO reporting in these studies is critically important. Only high-quality PRO reports can enhance the credibility of evidence on nursing intervention and effectively guide clinical practice.

In the reporting of PROs among the included studies, we have observed that 92.2% of the studies did not clearly specify whether PROs were a primary or secondary outcome in their abstracts (item P1b). This omission reduced the retrieval efficiency for readers and hindered further data analysis (59). 90.6% of the studies failed to fully state the hypothesis and domains related to PRO in the background (item P2b), which perhaps increase the risk of selective reporting in their reports. Khan et al. have observed the same findings (60).

Besides, we also found that the transparency of the PRO reports in the methodological part of the included trials was poor. Maring et al. even disclosed that many RCTs registered on clinicaltrials.gov regarding targeted therapy and/or immunotherapy for liver cancer patients reported PROs in their research protocols, but failed to report PROs in subsequent papers (21). According to the CONSORT-PRO extension (Item P6a), studies should report the validity and reliability of PROMs and a brief rationale for selecting the PROMs in trials. While just over 50% of the 64 studies provided or cited evidence on the validity and reliability of the PROMs. Most studies did not explain the reason for selecting PROMs. Specifically, we also observed regional preferences in the use of PROMs. For example, the SF-36 was used only in studies conducted in China. However, in these studies, researchers only cited the evidence for the validity of the PROMs without providing a brief rationale for selecting PROMs. This difference may be related to our search strategy, which only included studies published in Chinese or English in Chinese and English databases. It is also worth noting that adherence scores were lower in Chinese studies than in non-Chinese studies. We think that this is related to the predominance of non-confirmatory studies in Chinese studies and insufficient regulatory oversight of PROs. In the future, Chinese researchers should give full consideration to the reporting standards for PROs. Furthermore, only 9.4% of the studies explicitly described the method of collecting PRO data (item P6aiii). Different collection methods may affect the results and introduce potential biases (20). More than 90% of the studies failed to describe the handling methods for missing PRO data (Item P12a). This omission could raise a risk of selective outcome reporting. Missing PRO data due to disease progression or treatment discontinuation can introduce potential bias. Transparent reporting of the magnitude and reasons for missing data at each time point is crucial for clinicians to evaluate the potential bias of the PRO results. Notably, the Setting International Standards in Analyzing Patient-Reported Outcomes and Quality of Life Endpoints Data Consortium has published the guidelines, which provide recommendations on standardized statistical terminology and managing methods of missing PRO data (57). So, we advise that future RCTs using PRO as outcomes adhere to the guidelines for handling data.

Finally, the results and discussion section of PRO reports revealed some limitations. Specifically, 84.4% of studies reported only the total scores of PROMs, without fully presenting data or effect sizes for individual dimensions (Item 17a). The absence of data in dimensions impeded the development of detailed clinical decision-making at the domain-specific level. Failing to report effect sizes may cause readers to misinterpret the statistical or clinical significance of the research, weakening the persuasiveness of the results. Moreover, 90.6% of the studies completely omitted the evaluation of the limitations of the PROMs. while 75.0% did not discuss the generalizability or influence on clinical practice of the PRO results (Item P20/21). Most studies predominantly concentrated on describing PRO data, without a critical analysis of the PROMs’ suitability, sensitivity, or the coverage of dimensions. Future RCTs on targeted therapy and/or immunotherapy for liver cancer should analyze the limitations of PROMs in the discussion section, as recommended by guidelines. At the same time, it is advised to establish PRO databases for liver cancer patients to record regional discrepancies in responses to the same measures, thereby facilitating cross-cultural validation of these instruments. Regulatory authorities should draw on international experience to incorporate the assessment of PROMs into the approval requirements of clinical trials. Particularly for the innovative tools developed locally, evidence of equivalence to gold standard tools must be provided.

## 5 Limitation

In our scoping review, we conducted a systematic search and used the CONSORT-PRO checklist and the CONSORT 2010 standards to score the adherence of PRO reporting. However, there were still some limitations. Firstly, we only retrieved relevant RCTs in six databases. The limited databases may have resulted in the omission of some potential RCTs. Secondly, we only considered papers published in Chinese or English and excluded potentially eligible studies in other languages. Therefore, the majority of studies in this review originated from China (78.1%). Given that regional differences may exist in study design as well as in the selection and application of PROMs, this distribution may affect the generalizability of our findings. Therefore, caution is needed when extrapolating the results to other regions. Future systematic reviews should expand the scope of languages and databases to confirm the international applicability of these findings. Finally, we adhered the internationally recognized ICH E8(R1) framework to classify the types of included studies. However, we could not definitively determine whether some of the included nursing intervention trials and traditional Chinese medicine studies reported PRO should be classified as conventional confirmatory or exploratory studies. These studies were assigned to an “Other” category. The adoption of this “Other” classification strategy may have influenced the final results, and thus the findings of this study should be interpreted with caution. Future research is encouraged to report detailed study objectives and design more rigorously, so as to enable accurate stratification of study types and enhance the precision of the evidence.

## 6 Conclusions

We systematically evaluated the reporting quality of PRO in 64 RCTs of targeted therapy and/or immunotherapy for liver cancer. The results showed that the PROs reported in the included studies were highly concentrated on QoL. The most commonly used tool was the general EORTC QLQ-C30. The reporting of specific symptoms associated with targeted and/or immunotherapy, as well as liver cancer−specific symptoms, was inadequate. The application of disease-specific or treatment-specific PROMs was also used less frequently. Meanwhile, we also found that the integration of PROs with clinical endpoints was suboptimal in nursing-focused RCTs, and PRO data from medical studies were seldom used to guide nursing care practice. More importantly, the overall adherence scores to CONSORT-PRO of the included studies were low. Although confirmatory studies demonstrated higher scores than exploratory studies and other types of studies, there was considerable variation within this category. Common limitations included failure to clarify the role of PROs in abstracts, inadequate articulation of the hypothesis and domains about PRO in the background, low methodological transparency, particularly the handling methods for missing data, and incomplete interpretation of results. These issues undermine the interpretability of the findings and introduce potential bias into the research. In conclusion, future studies should prioritize the use of validated, standard disease-specific assessment tools to compensate for the limitations of generic instruments and strengthen the evaluation of treatment-related adverse events. Secondly, we advocate for the establishment of a collaborative framework that integrates medical, nursing, and patient perspectives, so that PRO data can effectively guide clinical decision−making and nursing practice. Furthermore, we also recommend that future studies, particularly confirmatory studies and nursing intervention or supportive care studies where PROs serve as primary outcomes, strictly adhere to the CONSORT−PRO guideline to enhance methodological transparency and reporting quality of PROs. Finally, all results obtained in this review are based solely on the 64 included studies, and future research should expand the search scope to enhance the generalizability of the evidence.

## Data Availability

The original contributions presented in the study are included in the article/**Supplementary Material**. Further inquiries can be directed to the corresponding author.

## Glossary

PROs: patient-reported outcomes
ISPOR: International Society for Pharmacoeconomics and Outcomes Research
FDA: U.S. Food and Drug Administration
PROMs: Patient-reported outcome measures
RCTs: randomized controlled trials
ISOQOL: International Society for Quality of Life Research
CONSORT-PRO: Consolidated Standards of Reporting Trials Extension for Patient-Reported Outcomes
QoL: quality of life
PRISMA-ScR: Preferred Reporting Items for Systematic Reviews and Meta-Analyses extension for scoping reviews
CNKI: China National Knowledge Infrastructure
CIK: Cytokine-Induced Killer
EORTC QLQ-C30: European Organization for Research and Treatment of Cancer Quality of Life Questionnaire
SF-36: Short Form-36 Health Survey
EORTC QLQ-HCC18: European Organization for Research and Treatment of Cancer Quality of Life Questionnaire-Hepatocellular Carcinoma 18
FACT-Hep: Functional Assessment of Cancer Therapy-Hepatobiliary
FACT-G: Functional Assessment of Cancer Therapy-General
EQ-5D: EuroQol Five Dimensions Questionnaire
QOL-LC: Quality-of-Life Questionnaire for Liver Cancer
DLQI: Dermatology Life Quality Index
HF-QoL: Hand-Foot Quality of Life questionnaire
PSQI: Pittsburgh Sleep Quality Index
VAS: Visual Analogue Scale
SAS: Self-Rating Anxiety Scale
SDS: Self-Rating Depression Scale
ESCA: Self-Care Ability Evaluation Scale
DBAS: Dysfunctional Beliefs and Attitudes on Sleep
NRS: Numerical Rating Scale
