# Supplementary material for: Evaluating patient-reported outcomes in randomized controlled trials of targeted therapy and/or immunotherapy for liver cancer: a scoping review
Source: Front Oncol. 2026 May 13;16:1770579. doi: 10.3389/fonc.2026.1770579 (PMC13216773; doi:10.3389/fonc.2026.1770579)
Supplement: Supplementary file 1 [file Table1.docx]

***Supplementary Material***

Appendix 1. The search strategies for each database

| Databases | Search strategies | Results |
| --- | --- | --- |
| PubMed  2025.04.16 | #1  "liver neoplasms"[MeSH Terms] | 206,914 |
|  | #2  "liver neoplasms"[MeSH Terms] OR "liver neoplasms"[Title/Abstract] OR "hepatocellular carcinoma"[Title/Abstract] OR "liver cancer"[Title/Abstract] OR "hepatic neoplasm"[Title/Abstract] OR "neoplasm hepatic"[Title/Abstract] OR "neoplasm hepatic"[Title/Abstract] OR "liver neoplasm"[Title/Abstract] OR "cancer of liver"[Title/Abstract] OR "hepatocellular cancer"[Title/Abstract] OR "hepatocellular cancer"[Title/Abstract] OR "cancer hepatocellular"[Title/Abstract] OR "hepatic cancer"[Title/Abstract] OR "cancer hepatic"[Title/Abstract] OR ((("Hepatic"[All Fields] OR "hepatophyta"[MeSH Terms] OR "hepatophyta"[All Fields] OR "hepatics"[All Fields]) AND "tumo"[All Fields]) AND "r"[Title/Abstract]) OR "hepatocellular neoplas*"[Title/Abstract] OR "hepatocellular neoplas*"[Title/Abstract] OR "hepatocellular tumorigenesis"[Title/Abstract] OR "liver cell tumor"[Title/Abstract] OR "liver cell tumour"[Title/Abstract] OR "liver neoplas*"[Title/Abstract] OR "liver tumorigenesis"[Title/Abstract] OR "neoplas* of the liver"[Title/Abstract] OR "neoplas* of the liver"[Title/Abstract] OR "neoplas* of the liver"[Title/Abstract] OR "tumor of the liver"[Title/Abstract] OR "tumor liver"[Title/Abstract] OR "tumour liver"[Title/Abstract] OR ((("Liver"[MeSH Terms] OR "Liver"[All Fields] OR "livers"[All Fields] OR "liver s"[All Fields]) AND "tumo"[All Fields]) AND "r"[Title/Abstract]) | 263,430 |
|  | #3  "gene therapy"[Title/Abstract] OR "immunotherapy"[Title/Abstract] OR "immune checkpoint inhibitors"[Title/Abstract] OR "immune checkpoint inhibitors"[Title/Abstract] OR "targeted therapy"[Title/Abstract] OR "molecular targeted therapy"[Title/Abstract] OR "TKIs"[Title/Abstract] OR "Lenvatinib"[Title/Abstract] OR "Sorafenib"[Title/Abstract] OR "Regorafenib"[Title/Abstract] OR "Cabozantinib"[Title/Abstract] OR "Ramucirumab"[Title/Abstract] OR "Bevacizumab"[Title/Abstract] OR "Atezolizumab"[Title/Abstract] OR "Durvalumab"[Title/Abstract] OR "Tremelimumab"[Title/Abstract] OR "Nivolumab"[Title/Abstract] OR "Ipilimumab"[Title/Abstract] OR "Sintilimab"[Title/Abstract] OR ("Iparomlimab"[Title/Abstract] AND "Tuvonralimab"[Title/Abstract]) | 323,468 |
|  | #4  "patient reported outcome measures"[MeSH Terms] | 18,256 |
|  | #5  (("patient s"[All Fields] OR "patients"[MeSH Terms] OR "patients"[All Fields] OR "patient"[All Fields] OR "patients s"[All Fields]) AND "report*"[All Fields] AND ("outcome"[All Fields] OR "outcomes"[All Fields])) OR ("patient* report*"[All Fields] AND ("outcome"[All Fields] OR "outcomes"[All Fields])) OR (("ego"[MeSH Terms] OR "ego"[All Fields] OR "self"[All Fields]) AND "report*"[All Fields]) OR "self report*"[All Fields] OR ("quality of life"[MeSH Terms] OR ("quality"[All Fields] AND "life"[All Fields]) OR "quality of life"[All Fields]) OR ("health status"[MeSH Terms] OR ("health"[All Fields] AND "status"[All Fields]) OR "health status"[All Fields]) OR (("health"[MeSH Terms] OR "health"[All Fields] OR "health s"[All Fields] OR "healthful"[All Fields] OR "healthfulness"[All Fields] OR "healths"[All Fields]) AND ("outcome"[All Fields] OR "outcomes"[All Fields])) OR ("depressed"[All Fields] OR "depression"[MeSH Terms] OR "depression"[All Fields] OR "depressions"[All Fields] OR "depression s"[All Fields] OR "depressive disorder"[MeSH Terms] OR ("depressive"[All Fields] AND "disorder"[All Fields]) OR "depressive disorder"[All Fields] OR "depressivity"[All Fields] OR "depressive"[All Fields] OR "depressively"[All Fields] OR "depressiveness"[All Fields] OR "depressives"[All Fields]) OR ("anxiety"[MeSH Terms] OR "anxiety"[All Fields] OR "anxieties"[All Fields] OR "anxiety s"[All Fields]) OR ("emoting"[All Fields] OR "emotion s"[All Fields] OR "emotions"[MeSH Terms] OR "emotions"[All Fields] OR "emotion"[All Fields] OR "emotional"[All Fields] OR "emotive"[All Fields]) OR ("social behavior"[MeSH Terms] OR ("social"[All Fields] AND "behavior"[All Fields]) OR "social behavior"[All Fields] OR "sociality"[All Fields] OR "social"[All Fields] OR "socialisation"[All Fields] OR "socialization"[MeSH Terms] OR "socialization"[All Fields] OR "socialise"[All Fields] OR "socialised"[All Fields] OR "socialising"[All Fields] OR "socialities"[All Fields] OR "socializations"[All Fields] OR "socialize"[All Fields] OR "socialized"[All Fields] OR "socializers"[All Fields] OR "socializes"[All Fields] OR "socializing"[All Fields] OR "socially"[All Fields] OR "socials"[All Fields]) OR ("psychosocial"[All Fields] OR "psychosocially"[All Fields]) OR ("psychologic"[All Fields] OR "psychological"[All Fields] OR "psychologically"[All Fields] OR "psychologization"[All Fields] OR "psychologized"[All Fields] OR "psychologizing"[All Fields]) OR ("distress"[All Fields] OR "distressed"[All Fields] OR "distresses"[All Fields] OR "distressful"[All Fields] OR "distressing"[All Fields]) OR (("social behavior"[MeSH Terms] OR ("social"[All Fields] AND "behavior"[All Fields]) OR "social behavior"[All Fields] OR "sociality"[All Fields] OR "social"[All Fields] OR "socialisation"[All Fields] OR "socialization"[MeSH Terms] OR "socialization"[All Fields] OR "socialise"[All Fields] OR "socialised"[All Fields] OR "socialising"[All Fields] OR "socialities"[All Fields] OR "socializations"[All Fields] OR "socialize"[All Fields] OR "socialized"[All Fields] OR "socializers"[All Fields] OR "socializes"[All Fields] OR "socializing"[All Fields] OR "socially"[All Fields] OR "socials"[All Fields]) AND "wellbeing"[All Fields]) OR ("social interaction"[MeSH Terms] OR ("social"[All Fields] AND "interaction"[All Fields]) OR "social interaction"[All Fields] OR ("social"[All Fields] AND "functioning"[All Fields]) OR "social functioning"[All Fields]) OR ("pain"[MeSH Terms] OR "pain"[All Fields]) OR "fatigu*"[All Fields] OR "HRQL"[All Fields] OR "QOL"[All Fields] OR ("hrqols"[All Fields] OR "quality of life"[MeSH Terms] OR ("quality"[All Fields] AND "life"[All Fields]) OR "quality of life"[All Fields] OR "hrqol"[All Fields]) OR (("diagnosis"[MeSH Subheading] OR "diagnosis"[All Fields] OR "symptoms"[All Fields] OR "diagnosis"[MeSH Terms] OR "symptom"[All Fields] OR "symptom s"[All Fields] OR "symptomes"[All Fields]) AND ("distress"[All Fields] OR "distressed"[All Fields] OR "distresses"[All Fields] OR "distressful"[All Fields] OR "distressing"[All Fields])) OR ("symptom burden"[MeSH Terms] OR ("symptom"[All Fields] AND "burden"[All Fields]) OR "symptom burden"[All Fields]) OR ("symptom burden"[MeSH Terms] OR ("symptom"[All Fields] AND "burden"[All Fields]) OR "symptom burden"[All Fields]) OR ("functional status"[MeSH Terms] OR ("functional"[All Fields] AND "status"[All Fields]) OR "functional status"[All Fields]) OR ("sexual behavior"[MeSH Terms] OR ("sexual"[All Fields] AND "behavior"[All Fields]) OR "sexual behavior"[All Fields]) OR "functional*"[All Fields] OR "functional*"[All Fields] OR ("physiologies"[All Fields] OR "physiology"[MeSH Subheading] OR "physiology"[All Fields] OR "physiology"[MeSH Terms]) | 19,821,402 |
|  | #6  #4 OR #5 | 19,821,412 |
|  | #7  #2 AND #3 | 19,167 |
|  | #8  #7 AND #6 | 10,784 |
|  | #9  randomized controlled trials as topic[MeSH Terms] | 185,088 |
|  | #10  "randomized controlled trials as topic"[MeSH Terms] OR "randomized controlled trial"[Title/Abstract] OR (("random allocation"[MeSH Terms] OR ("random"[All Fields] AND "allocation"[All Fields]) OR "random allocation"[All Fields] OR "randomization"[All Fields] OR "randomized"[All Fields] OR "random"[All Fields] OR "randomisation"[All Fields] OR "randomisations"[All Fields] OR "randomise"[All Fields] OR "randomised"[All Fields] OR "randomising"[All Fields] OR "randomizations"[All Fields] OR "randomize"[All Fields] OR "randomizes"[All Fields] OR "randomizing"[All Fields] OR "randomness"[All Fields] OR "randoms"[All Fields]) AND "ed"[Title/Abstract]) OR "randomly"[Title/Abstract] OR "RCT"[Title/Abstract] | 749,670 |
|  | #11  #8 AND#10 | 271 |
| Web of Science  2025.04.17 | #1  ((TS=("Liver Neoplasms")) OR AB=("Liver Neoplasms" OR "hepatocellular carcinoma" OR "liver cancer?" OR "Hepatic Neoplasm?" OR "Neoplasm?, Hepatic" OR "Neoplasm?, Liver" OR "Liver Neoplasm?" OR "Cancer of Liver" OR "Hepatocellular Cancer?" OR "Cancer of the Liver" OR "Cancer?, Hepatocellular" OR "Hepatic Cancer?" OR "Cancer?, Hepatic" OR "hepatic tumo?r" OR "hepatocellular neoplas*" OR "hepatocellular tumo?r" OR "hepatocellular tumorigenesis" OR "hepatocyte tumorigenesis" OR "liver cell tumor" OR "liver cell tumour" OR "liver neoplas*" OR "liver tumorigenesis" OR "liver tumorigenesis" OR "neoplastic hepatocellular" OR "neoplastic liver" OR "tumor of the liver" OR "tumor, liver" OR "tumour, liver" OR "liver tumo?r")) and Preprint Citation Index (Exclude – Database) | 304,574 |
|  | #2  AB=("Gene therapy" OR "immunotherapy" OR "immunotherapy" OR "ICIs" OR "targeted therapy" OR "Molecular targeted therapy" OR "Lenvatinib" OR "Sorafenib" OR "Regorafenib" OR "Cabozantinib" OR "Ramucirumab" OR "Bevacizumab" OR "Atezolizumab" OR "Durvalumab" OR "Tremelimumab" OR "Nivolumab" OR "Ipilimumab" OR "Sintilimab" OR "Iparomlimab and Tuvonralimab") and Preprint Citation Index (Exclude – Database) | 280,839 |
|  | #3  (TS=("Patient Reported Outcome Measures")) OR AB=("Patient Report* Outcome?" OR "Patient*-report* Outcome?" OR "Self report*" OR "Self-report*" OR "Self-report*" OR "health status" OR "health outcomes" OR "depression" OR "anxiety" OR "emotional" OR "emotional" OR "social" OR "psychosocial" OR "psychological" OR "distress" OR "social functioning" OR "social wellbeing" OR "pain" OR "fatigu*" OR "HRQL" OR "QOL" OR "HRQOL" OR "symptom distress" OR "symptom burden" OR "symptom assessment" OR "functional status " OR "sexual behavior" OR "functional*" OR "functioned" OR "physiology") and Preprint Citation Index (Exclude – Database) | 7,822,552 |
|  | #4  AB=("randomized controlled trial" OR "random?ed" OR "randomly" OR "RCT") and Preprint Citation Index (Exclude – Database) | 775,975 |
|  | #5  #4 AND #3 AND #2 AND #1 and Preprint Citation Index (Exclude – Database) | 46 |
| Embase  2025.04.17 | #1  'liver tumor'/exp/mj | 232,753 |
|  | #2  'liver neoplasms':ab,ti OR 'hepatocellular carcinoma':ab,ti OR 'liver cancer?':ab,ti OR 'hepatic neoplasm?':ab,ti OR 'neoplasm?, hepatic':ab,ti OR 'neoplasm?, liver':ab,ti OR 'liver neoplasm?':ab,ti OR 'cancer of liver':ab,ti OR 'hepatocellular cancer?':ab,ti OR 'cancer of the liver':ab,ti OR 'cancer?, hepatocellular':ab,ti OR 'hepatic cancer?':ab,ti OR 'cancer?, hepatic':ab,ti OR 'hepatic tumo?r':ab,ti OR 'hepatocellular neoplas*':ab,ti OR 'hepatocellular tumo?r':ab,ti OR 'hepatocellular tumorigenesis':ab,ti OR 'hepatocyte tumorigenesis':ab,ti OR 'liver cell tumor':ab,ti OR 'liver cell tumour':ab,ti OR 'liver neoplas*':ab,ti OR 'liver tumorigenesis':ab,ti OR 'neoplastic hepatocellular':ab,ti OR 'neoplastic liver':ab,ti OR 'tumor of the liver':ab,ti OR 'tumor, liver':ab,ti OR 'tumour, liver':ab,ti OR 'liver tumo?r':ab,ti | 194,390 |
|  | #3  #1 OR #2 | 293,083 |
|  | #4  'gene therapy':ab,ti OR 'immunotherapy':ab,ti OR 'icis':ab,ti OR 'targeted therapy':ab,ti OR 'molecular targeted therapy':ab,ti OR 'lenvatinib':ab,ti OR 'sorafenib':ab,ti OR 'regorafenib':ab,ti OR 'cabozantinib':ab,ti OR 'ramucirumab':ab,ti OR 'bevacizumab':ab,ti OR 'atezolizumab':ab,ti OR 'durvalumab':ab,ti OR 'tremelimumab':ab,ti OR 'nivolumab':ab,ti OR 'ipilimumab':ab,ti OR 'sintilimab':ab,ti OR ('iparomlimab':ab,ti AND 'tuvonralimab':ab,ti) | 440,438 |
|  | #5  'patient-reported outcome'/exp | 72,297 |
|  | #6  'patient report* outcome?':ab,ti OR 'patient*-report* outcome?':ab,ti OR 'self report*':ab,ti OR 'self-report*':ab,ti OR 'health status':ab,ti OR 'health outcomes':ab,ti OR 'depression':ab,ti OR 'anxiety':ab,ti OR 'emotional':ab,ti OR 'social':ab,ti OR 'psychosocial':ab,ti OR 'psychological':ab,ti OR 'distress':ab,ti OR 'social functioning':ab,ti OR 'social wellbeing':ab,ti OR 'pain':ab,ti OR 'fatigu*':ab,ti OR 'hrql':ab,ti OR 'qol':ab,ti OR 'hrqol':ab,ti OR 'symptom distress':ab,ti OR 'symptom burden':ab,ti OR 'symptom assessment':ab,ti OR 'functional status':ab,ti OR 'sexual behavior':ab,ti OR 'functional*':ab,ti OR 'functioned':ab,ti OR 'physiology':ab,ti | 6,317,880 |
|  | #7  #5 OR #6 | 6,335,449 |
|  | #8  'randomized controlled trial':ab,ti OR 'random?ed':ab,ti OR 'randomly':ab,ti OR 'rct':ab,ti | 777,127 |
|  | #9  #3 AND #4 AND #7 AND #8 | 67 |
| China National Knowledge Infrastructure (CNKI)  2025.04.17 | SU=('Liver Cancer' + 'hepatocellular carcinoma' + 'Primary Liver Cancer') AND SU=('Gene Therapy' + 'Immunotherapy' + 'Targeted Therapy' + 'Immune Checkpoint Inhibitors' + Sorafenib + Lenvatinib + Regorafenib + 'TK' + 'PD-L1' + 'PD-1' + Cabozantinib + Ramucirumab + Atezolizumab + Bevacizumab + Durvalumab) AND FT=('Patient-Reported Outcomes' + 'PRO' + Self-Reported + Patient-Reported + Outcomes + Assessment + Tools + 'Outcome Measures' + Questionnaires + 'Quality of Life' + Pain + Nutrition + Fatigue + Anxiety + Depression + 'Physical Function' + 'Physical Activity' + Sleep + Self-Efficacy) AND AB=(Randomized + 'RCT') | 568 |
| WANFANG DATA  2025.04.17 | Subject:(Hepatocellular carcinoma OR Liver cancer) AND All fields:(Self-reported OR Patient-reported OR Outcome OR Measurement OR Tool OR Scale OR Questionnaire OR Score OR PRO OR PROM OR Quality of life OR Pain OR Nutrition OR Fatigue OR Anxiety OR Depression OR Physical function OR Physical performance OR Sleep OR Self-efficacy) AND Abstract:(gene therapy OR immunotherapy OR targeted therapy OR immune checkpoint inhibitors OR TKI OR PD-L1 OR PD-1 OR Cabozantinib OR ICIs OR Lenvatinib OR Sorafenib OR Regorafenib OR Ramucirumab OR Bevacizumab OR Atezolizumab OR Durvalumab OR Tremelimumab OR Nivolumab OR Ipilimumab OR Sintilimab OR Iparomlimab and Tuvonralimab) AND Abstract:(Randomized OR RCT) | 510 |
| VIP  2025.04.17 | M=(liver cancer OR hepatocellular carcinoma) AND U=(Self-reported OR patient-reported OR outcome OR measurement OR tool OR scale OR questionnaire OR score OR PRO OR PROM OR quality of life OR pain OR nutrition OR fatigue OR anxiety OR Depression OR physical function OR physical strength OR sleep OR self-efficacy) AND R=(gene therapy OR immunotherapy OR targeted therapy OR immune checkpoint inhibitors OR TKI OR PD-L1 OR PD-1 OR Cabozantinib OR ICIs OR Lenvatinib OR Sorafenib OR Regorafenib OR Ramucirumab OR Bevacizumab OR Atezolizumab OR Durvalumab OR Tremelimumab OR Nivolumab OR Ipilimumab OR Sintilimab OR Iparomlimab and Tuvonralimab) AND R=(random OR RCT) | 446 |
