# Supplementary material for: Evaluating patient-reported outcomes in randomized controlled trials of targeted therapy and/or immunotherapy for liver cancer: a scoping review
Source: Front Oncol. 2026 May 13;16:1770579. doi: 10.3389/fonc.2026.1770579 (PMC13216773; doi:10.3389/fonc.2026.1770579)
Supplement: Supplementary file 2 [file Table2.docx]

1. **Supplementary Tables**

Table 1. Characteristics and key results of included studies (*N*=64)

| First author | Pub. year | Study center | Country | Study type | Sample size/Male | Treatment types | Treatment arms | PROs | PROMs | Outcome | CONSORT-PRO Score |
| --- | --- | --- | --- | --- | --- | --- | --- | --- | --- | --- | --- |
| A. L. Cheng^1^ | 2009 | Multiple | China | C | 226/193 | Target therapy VS placebo | Sorafenib VS placebo | Physical well-being | FACT-Hep | SO | 6.0 |
| J. Bruix^2^ | 2017 | Multiple | France | C | 573/504 | Target therapy VS placebo | Regorafenib VS placebo | HRQoL | EQ-5D and EQ-VAS | SO | 3.0 |
| T. Meyer^3^ | 2017 | Single | UK | C | 313/277 | Target therapy plus Chemotherapy VS placebo plus Chemotherapy | Sorafenib plus Chemotherapy VS placebo plus Chemotherapy | QoL | EORTC QLQ-C30 and QLQ-HCC18 and EQ-5D | SO | 6.5 |
| V. Vilgrain^4^ | 2017 | Multiple | France | C | 459/414 | radiation therapy VS Target therapy | Radiation therapy VS Sorafenib | QoL | EORTC QLQ-C30 and QLQ-HCC18 | SO | 5.5 |
| L. Rimassa^5^ | 2018 | Multiple | Italy | C | 340/306 | Target therapy VS placebo | Tivantinib VS placebo | QoL | FACT-Hep EQ-5D | SO | 4.5 |
| P. K. H. Chow^6^ | 2018 | Multiple | Singapore | C | 360/299 | radiation therapy VS Target therapy | radiation therapy VS Sorafenib | HRQoL | EQ-5D | SO | 8.5 |
| J. L. Jouve^7^ | 2019 | Multiple | France | C | 323/298 | Target therapy plus lipid lowering drugs VS Target therapy | Sorafenib plus Pravastatin VS Sorafenib | QoL | EORTC QLQ-C30 and FACT-Hep | SO | 9.0 |
| R. S. Finn^8^ | 2020 | Multiple | America | C | 501/414 | Target therapy plus Immunotherapy VS Target therapy | Bevacizumab plus Atezolizumab VS Sorafenib | QoL | EORTC QLQ-C30 | SO | 4.0 |
| A. Vogel^9^ | 2021 | Multiple | Germany | C | 931 | Target therapy VS Target therapy | Lenvatinib VS Sorafenib | QoL | EORTC QLQ-C30 and QLQ-HCC18 | SO | 13.0 |
| B. Y. Ryoo^10^ | 2021 | Single | France | C | 410 | Immunotherapy VS placebo | Pembrolizumab VS placebo | QoL | EORTC QLQ-C30 and QLQ-HCC18 | SO | 11.0 |
| P. R. Galle^11^ | 2021 | Multiple | Germany | C | 454 | Target therapy plus Immunotherapy VS Target therapy | Bevacizumab plus Atezolizumab VS Sorafenib | QoL | EORTC QLQ-C30 and QLQ-HCC18 | PO | 12.5 |
| G. K. Abou-Alfa^12^ | 2022 | Multiple | America | C | 1171 | Immunotherapy plus Immunotherapy VS Immunotherapy VS Target therapy | Tislelizumab plus Durvalumab VS Durvalumab VS Sorafenib | QoL | EORTC QLQ-C30 | SO | 3.5 |
| B. Sangro^13^ | 2024 | Multiple | Spain | C | 1171 | Immunotherapy plus Immunotherapy VS Immunotherapy VS Target therapy | Tislelizumab plus Durvalumab VS Durvalumab VS Sorafenib | QoL | EORTC-QLQ-C30 and QLQ-HCC18 | SO | 14.0 |
| B. Sangro^14^ | 2025 | Multiple | Spain | C | 616 | Target therapy plus Immunotherapy plus Chemotherapy VS Immunotherapy plus placebo VS placebo | durvalumab plus Bevacizumab plus Chemotherapy VS durvalumab plus placebo VS placebo | QoL | EORTC-QLQ-C30 and QLQ-HCC18 | SO | 4.0 |
| D. Koeberle^15^ | 2014 | Multiple | Switzerland | E | 106/88 | Target therapy plus Target therapy VS Target therapy | Sorafenib plus Everolimus VS Sorafenib | QoL | FACT-Hep | SO | 5.0 |
| C. X. Qiao^16^ | 2015 | Single | China | E | 40/33 | Target therapy plus traditional Chinese medicine VS Target therapy | Sorafenib plus traditional Chinese medicine VS Sorafenib | QoL | FACT-Hep | SO | 5.5 |
| X.L. Chen^17^ | 2015 | Single | China | E | 90/50 | Target therapy plus Chemotherapy plus radiation therapy VS Target therapy plus Chemotherapy | gefitinib plus Chemotherapy plus radiation therapy VS gefitinib plus Chemotherapy | QoL | EORTC QLQ-C30 | PO | 9.0 |
| H.M. Liu^18^ | 2015 | Single | China | E | 156 | Immunotherapy plus Interventional treatment VS Immunotherapy | CIK plus Interventional treatment VS CIK | QoL | EORTC QLQ-C30 | PO | 7.0 |
| H.X. Zhu^19^ | 2015 | Single | China | E | 90/50 | Target therapy plus Chemotherapy VS Chemotherapy | gefitinib plus Chemotherapy VS Chemotherapy | QoL | EORTC QLQ-C30 | PO | 6.0 |
|  |  |  |  |  |  |  |  | Pain | VAS |  |  |
| C.X. Qiao^20^ | 2017 | Single | China | E | 80/73 | Target therapy plus Immunotherapy VS Target therapy | CIK plus Sorafenib VS Sorafenib | QoL | FACT-Hep | SO | 7.5 |
| S.P. Jiang^21^ | 2018 | Single | China | E | 48/27 | Target therapy plus Immunotherapy VS Target therapy | Sorafenib plus CIK VS Sorafenib | QoL | FACT-Hep | PO | 6.5 |
| J.X. Huang^22^ | 2021 | Single | China | E | 62/42 | Immunotherapy plus Chemotherapy VS Chemotherapy | Camrelizumab plus Chemotherapy VS Chemotherapy | QoL | QOL-LC | SO | 6.0 |
|  |  |  |  |  |  |  |  | Pain | VAS |  |  |
| P.T. Zhai^23^ | 2021 | Single | China | E | 100/52 | Target therapy plus Interventional treatment VS Interventional treatment | Apatinib plus Interventional treatment VS Interventional treatment | QoL | SF-36 | SO | 6.0 |
| X.Y. Wang^24^ | 2021 | Single | China | E | 120/81 | Targeted combined immunotherapy plus Chemotherapy plus Psychiatric Nursing VS Chemotherapy plus Routine nursing mode | Sorafenib plus Chemotherapy plus Psychiatric Nursing VS Chemotherapy plus Routine nursing mode | QoL | SF-36 | SO | 6.5 |
| Z.F. Deng^25^ | 2022 | Single | China | E | 100/58 | Target therapy plus Immunotherapy VS Target therapy plus Immunotherapy | Lenvatinib plus Camrelizumab VS Sorafenib plus Camrelizumab | QoL | SF-36 | SO | 6.0 |
| W.Q. Li^26^ | 2022 | Single | China | E | 100/67 | Target therapy plus Immunotherapy VS Target therapy | Lenvatinib plus Sintilimab VS Lenvatinib | QoL | WHOQOL-BREF | SO | 6.0 |
| P. Ma^27^ | 2022 | Single | China | E | 58/34 | Target therapy plus Chemotherapy VS Chemotherapy | Lenvatinib plus Chemotherapy VS Chemotherapy | Functional Assessment of Cancer Therapy | FACT-G | SO | 4.5 |
| F.Q. Zhang^28^ | 2022 | Single | China | E | 80/63 | Target therapy plus Chemotherapy VS Chemotherapy | Lenvatinib plus Chemotherapy VS Chemotherapy | QoL | SF-36 | SO | 6.0 |
| F. Cao^29^ | 2023 | Single | China | E | 40/25 | Target therapy plus Immunotherapy plus Chemotherapy VS Chemotherapy | Lenvatinib plus Sorafenib plus Camrelizumab plus Chemotherapy VS Chemotherapy | QoL | SF-36 | SO | 5.5 |
| L. Di^30^ | 2023 | Single | China | E | 104/62 | Target therapy plus Immunotherapy VS Target therapy | Lenvatinib plus Toripalimab VS Lenvatinib | QoL | EORTC QLQ-C30 | SO | 5.0 |
| Y.S. Fu^31^ | 2023 | Single | China | E | 102/58 | Immunotherapy plus Interventional treatment VS Target therapy | Sintilimab plus Interventional treatment VS Interventional treatment | QoL | SF-36 | SO | 7.0 |
| Z.C. Hu^32^ | 2023 | Single | China | E | 82/48 | Target therapy plus Immunotherapy plus Interventional treatment VS Target therapy plus Interventional treatment | Sorafenib plus NK* cell immunotherapy plus Interventional treatment VS Sorafenib plus Interventional treatment | QoL | FACT-G | SO | 6.0 |
| Q.H. Liao^33^ | 2023 | Single | China | E | 70/39 | Target therapy plus Immunotherapy plus Chemotherapy plus Chemotherapy VS Target therapy plus Immunotherapy plus Chemotherapy | Lenvatinib plus Camrelizumab plus Chemotherapy plus Chemotherapy VS Lenvatinib plus Camrelizumab plus Chemotherapy | QoL | EORTC QLQ-C30 | SO | 6.0 |
| H.H. Ye^34^ | 2023 | Single | China | E | 68 | Immunotherapy plus Chemotherapy VS Chemotherapy | Tislelizumab plus Chemotherapy VS Chemotherapy | QoL | FACT-G | SO | 6.0 |
| Z.H. Liu^35^ | 2024 | Single | China | E | 62 | Target therapy plus Chemotherapy VS Chemotherapy | Sorafenib plus Chemotherapy VS Chemotherapy | Pain | VAS | SO | 4.0 |
| Y. Qu^36^ | 2024 | Single | China | E | 90 | Immunotherapy plus surgery VS surgery | CIK plus surgery VS surgery | QoL | SF-36 | SO | 5.0 |
| X.L. Wu^37^ | 2024 | Single | China | E | 108 | Target therapy plus Immunotherapy plus Chemotherapy VS Target therapy plus Chemotherapy | Toripalimab plus Lenvatinib plus Chemotherapy VS Lenvatinib plus Chemotherapy | QOL | FACT-G | SO | 5.0 |
| F. Yang^38^ | 2024 | Single | China | E | 92 | Immunotherapy plus Immunotherapy plus Chemotherapy VS Immunotherapy plus Chemotherapy | CIK plus Pembrolizumab plus Chemotherapy VS CIK plus Chemotherapy | QoL | EORTC QLQ-C30 | SO | 5.5 |
| L.F. Yang^39^ | 2024 | Single | China | E | 64 | Target therapy plus Immunotherapy VS Target therapy | Camrelizumab plus Lenvatinib VS Lenvatinib | QOL | EORTC QLQ-C30 | SO | 6.0 |
| X. Yin^40^ | 2024 | Single | China | E | 102 | Target therapy plus Target therapy VS Target therapy | Sorafenib plus Ramucirumab VS Sorafenib | QoL | SF-36 | SO | 4.5 |
| G.H. Zhong^41^ | 2024 | Single | China | E | 58 | Target therapy plus Immunotherapy plus Chemotherapy plus Chemotherapy VS Chemotherapy plus Chemotherapy | Bevacizumab plus Sintilimab plus Chemotherapy plus Chemotherapy VS Chemotherapy plus Chemotherapy | Sleep Quality | PSQI | SO | 5.0 |
| Y. Chen^42^ | 2025 | Single | China | E | 60 | Target therapy plus Target therapy VS Target therapy | Sorafenib plus Apatinib VS Sorafenib | QoL | SF-36 | SO | 7.5 |
| H. Fang^43^ | 2025 | Single | China | E | 300 | Target therapy plus Chemotherapy VS Target therapy | Sorafenib plus Chemotherapy VS Sorafenib | QoL | SF-36 | SO | 5.0 |
| J.Y. Yan^44^ | 2025 | Single | China | E | 112 | Immunotherapy plus Interventional treatment VS Interventional treatment | Atezolizumab plus Interventional treatment VS Interventional treatment | QoL | EORTC QLQ-C30 | SO | 5.0 |
| D.Y. Zhu^45^ | 2025 | Single | China | E | 62 | Immunotherapy plus Chemotherapy VS Chemotherapy | Camrelizumab plus Chemotherapy VS Chemotherapy | QoL | QLACS | SO | 4.5 |
| Z. Ren^46^ | 2015 | Multiple | China | O | 871/745 | Target therapy plus BSC* VS Target therapy | Sorafenib plus BSC VS Sorafenib | Hand-foot skin reaction and QoL | HF-QoL | SO | 6.5 |
| J. Guo^47^ | 2016 | Single | China | O | 60/31 | Immunotherapy plus Optimize nursing intervention VS Immunotherapy plus Routine nursing mode | CIK plus Optimize nursing intervention VS CIK plus Routine nursing mode | Anxiety | SAS | PO | 7.5 |
|  |  |  |  |  |  |  |  | Depression | SDS |  |  |
| H.M. Liu^48^ | 2016 | Single | China | O | 156/95 | Immunotherapy plus cognitive intervention VS Immunotherapy plus Routine nursing mode | CIK plus Cognitive intervention VS CIK plus Routine nursing mode | QoL | EORTC QLQ-C30 | PO | 9.0 |
|  |  |  |  |  |  |  |  | Pain | VAS |  |  |
| J. Kuang^49^ | 2020 | Single | China | O | 96/58 | Target therapy plus surgery plus ERAS* VS Target therapy plus surgery plus Routine nursing mode | Sorafenib plus surgery plus ERAS* VS Sorafenib plus surgery plus Routine nursing mode | QoL | EORTC QLQ-C30 | SO | 3.0 |
| J.Z. Chen^50^ | 2021 | Single | China | O | 58/31 | Target therapy plus High quality nursing VS Target therapy plus Routine nursing mode | No reported | QoL | GQOLI-74 | SO | 6.5 |
|  |  |  |  |  |  |  |  | Sleep Quality | PSQI |  |  |
| Y.J. Deng^51^ | 2021 | Single | China | O | 66/47 | Target therapy plus traditional Chinese medicine VS Target therapy | Sorafenib plus traditional Chinese medicine VS Sorafenib | QoL | QOL-LC | PO | 5.5 |
| S. Wang^52^ | 2021 | Single | China | O | 100/54 | Target therapy plus humanistic nursing plus Narrative Nursing VS Target therapy plus humanistic nursing | Sorafenib plus humanistic nursing plus Narrative Nursing plus VS Sorafenib plus humanistic nursing | Well-being | Campbell IWB | PO | 5.0 |
|  |  |  |  |  |  |  |  | Negative emotions | MCMQ |  |  |
|  |  |  |  |  |  |  |  | QoL | EORTC QLQ-C30 |  |  |
| Y.L. Yang^53^ | 2022 | Single | China | O | 140/90 | Target therapy plus CNP* VS Target therapy plus Routine nursing mode | Sorafenib plus CNP VS Sorafenib plus Routine nursing mode | Sleep Quality | PSQI | PO | 5.5 |
|  |  |  |  |  |  |  |  | QoL | EORTC QLQ-C30 |  |  |
| Y.F. Zhang^54^ | 2022 | Single | China | O | 88/43 | Target therapy plus Chemotherapy VS Target therapy | Sorafenib plus Chemotherapy VS Sorafenib | Sleep Quality | PSQI | PO | 5.0 |
|  |  |  |  |  |  |  |  | Anxiety | SAS |  |  |
|  |  |  |  |  |  |  |  | Depression | SDS |  |  |
| Y. Liu^55^ | 2023 | Single | China | O | 120/82 | Target therapy plus Chemotherapy plus Orem's Self-Care Model VS Target therapy plus Chemotherapy plus Routine nursing mode | Sorafenib plus Chemotherapy plus Orem's Self-Care Model VS Sorafenib plus Chemotherapy plus Routine nursing mode | SCA | ESCA | PO | 7.0 |
|  |  |  |  |  |  |  |  | Anxiety | SAS |  |  |
|  |  |  |  |  |  |  |  | Depression | SDS |  |  |
| L.Q. Yao^56^ | 2023 | Single | China | O | 80/51 | Target therapy plus Immunotherapy plus Exercise - Psychological - Sleep Nursing Intervention plus Aromatherapy VS Target therapy plus Immunotherapy plus Routine nursing mode | No reported | Sleep Quality | PSQI and DBAS | PO | 8.5 |
|  |  |  |  |  |  |  |  | QoL | EORTC QLQ-C30 |  |  |
|  |  |  |  |  |  |  |  | Patient satisfaction | Self-made patient satisfaction survey form |  |  |
| D. Yu^57^ | 2023 | Single | China | O | 112/69 | Immunotherapy plus traditional Chinese medicine VS Immunotherapy | Nivolumab/Sintilimab/Camrelizumab plus traditional Chinese medicine VS Nivolumab/Sintilimab/Camrelizumab | Pain | NRS | SO | 5.5 |
| Y.H. Zhu^58^ | 2023 | Single | China | O | 70/53 | Immunotherapy plus traditional Chinese medicine VS Immunotherapy | No reported | Dermatology Life Quality | DLQI | SO | 5.5 |
| L.J. Deng^59^ | 2024 | Single | China | O | 60 | Target therapy plus Cloud hospital nursing management model VS Target therapy plus Routine nursing mode | No reported | Medication Adherence | MMAS-8 | SO | 6.5 |
|  |  |  |  |  |  |  |  | Managerial self-efficacy | SUPPH |  |  |
|  |  |  |  |  |  |  |  | SCA | ESCA |  |  |
| D.Y. Jiang^60^ | 2024 | Single | China | O | 80 | Immunotherapy plus High quality nursing VS Immunotherapy plus Routine nursing mode | No reported | Anxiety | SAS | PO | 7.0 |
|  |  |  |  |  |  |  |  | Depression | SDS |  |  |
|  |  |  |  |  |  |  |  | Cognitive function | MMSE |  |  |
|  |  |  |  |  |  |  |  | Sleep Quality | PSQI |  |  |
|  |  |  |  |  |  |  |  | QoL | SF-36 |  |  |
| P. Lu^61^ | 2024 | Single | China | O | 120 | Target therapy plus Psychological intervention based on dignity therapy VS Target therapy plus Routine nursing mode | No reported | Medication Adherence | MMAS-8 | PO | 7.0 |
|  |  |  |  |  |  |  |  | Patient Dignity | PDI |  |  |
|  |  |  |  |  |  |  |  | Self-Perceived Burden | SPBS |  |  |
|  |  |  |  |  |  |  |  | QoL | EORTC QLQ-C30 |  |  |
| P.Y. Yang^62^ | 2024 | Single | China | O | 60 | Target therapy plus traditional Chinese medicine plus traditional Chinese medicine VS Target therapy plus traditional Chinese medicine | Sorafenib plus traditional Chinese medicine plus traditional Chinese medicine VS Sorafenib plus traditional Chinese medicine | QoL | SF-36 | SO | 4.5 |
| Y. Yao^63^ | 2024 | Single | China | O | 93 | Target therapy plus traditional Chinese medicine VS Target therapy | Lenvatinib plus traditional Chinese medicine VS Lenvatinib | QoL | EORTC QLQ-HCC18 | SO | 4.0 |
| K. Zhou^64^ | 2024 | Single | China | O | 61 | Target therapy plus Immunotherapy plus traditional Chinese medicine VS Target therapy plus Immunotherapy | Bevacizumab plus Tislelizumab plus traditional Chinese medicine VS Bevacizumab plus Tislelizumab | QoL | EORTC QLQ-C30 | SO | 5.5 |
|  |  |  |  |  |  |  |  | Pain | VAS |  |  |
| Explanations: | | | | | | | | | | | |
| PROs, Patient-Reported Outcomes; PROMs, Patient-Reported Outcomes Measures; SO, Secondary Outcome; PO, Primary Outcome; CONSORT, Consolidated Standards of Reporting Trials; BSC, Best Supportive Care; ERAS, Enhanced Recovery After Surgery; CNP, Clinical Cursing Pathways; CIK, Cytokine-Induced Killer; NK, Natural Killer; QoL, Quality of Life; HRQoL, Health-Related Quality of Life; SCA, Self-Care Agency; FACT-Hep, Functional Assessment of Cancer Therapy-Hepatobiliary; HF-QoL, Hand-Foot Skin Reaction and Quality of Life Questionnaire; EORTC QLQ-C30, European Organization for Research and Treatment of Cancer Quality of Life Questionnaire; VAS, Visual Analogue Scale; EQ-5D, EuroQol Five Dimensions Questionnaire; QLQ-HCC18, Quality of Life Questionnaire-Hepatocellular Carcinoma 18; SAS, Self-Rating Anxiety Scale; SDS, Self-rating depression scale; GQOLI-74, Generic Quality of Life Inventory-74; PSQI, Pittsburgh sleep quality index; QoL-LC, Quality Of Life-Liver; SF-36, Short Form-36 Health Survey; IWB, index of well-being; MCMQ, Medical Coping Modes Questionnaire; WHOQOL-BREF, World Health Organization Quality of Life - Brief Version; FACT-G, Functional Assessment of Cancer Therapy-General; ESCA, Self-Care Ability Scale; DBAS, Dysfunctional Beliefs and Attitudes on Sleep; NRS, Numerical Rating Scale; DLQI, Dermatology Life Quality Index; MMAS-8, Morisky Medication Adherence Scale-8; SUPPH, strategies used by people to promote health; MMSE, Minimum Mental State Examination; PDI, Patient Dignity Inventory; SPBS, Self-Perceived Burden Scale; QLACS, Quality of Life in Adult Cancer Survivors. | | | | | | | | | | | |
| Study Type: C, Confirmatory: Phase III trials or large-scale registrational trials designed to support licensing or establish definitive efficacy. E, Exploratory: Phase I/II trials or smaller-scale efficacy studies designed to explore preliminary efficacy or estimate dosing. O, Other: Nursing interventions, traditional Chinese medicine studies, supportive care evaluations, and mechanism-focused studies that do not conform to the traditional phase classification. | | | | | | | | | | | |

Table 2. The characteristics of patient-reported outcome in the included studies (*N*=64)

| PROs | PROMs | *N*（%） | PROs | PROMs | *N*（%） |
| --- | --- | --- | --- | --- | --- |
| QoL | EORTC QLQ-C30 | 17(26.6) | Sleep Quality | PSQI | 5(7.8) |
|  | SF-36 | 12(18.8) |  | PSQI、DBAS | 1(1.6) |
|  | FACT-Hep | 4(6.3) | Pain | VAS | 5(7.8) |
|  | FACT-G | 3(4.7) |  | NRS | 1(1.6) |
|  | QoL-LC | 2(3.1) | Anxiety | SAS | 4(6.3) |
|  | EORTC QLQ-HCC18 | 1(1.6) | Depression | SDS | 4(6.3) |
|  | WHOQOL-BREF | 1(1.6) | SCA | ESCA | 2(3.1) |
|  | QLACS | 1(1.6) | Dermatology life quality | DLQI | 1(1.6) |
|  | GQOLI-74 | 1(1.6) | Hand-foot skin reaction and QoL | HF-QoL | 1(1.6) |
|  | EORTC QLQ-C30 plus QLQ-HCC18 | 6(9.4) | Managerial self-efficacy | SUPPH | 1(1.6) |
|  | EORTC QLQ-C30 plus QLQ-HCC18 plus EQ-5D | 1(1.6) | Well-being | Campbell IWB | 1(1.6) |
|  | EORTC QLQ-C30 plus FACT-Hep | 1(1.6) | Patient dignity | PDI | 1(1.6) |
|  | FACT-Hep plus EQ-5D | 1(1.6) | Functional assessment of cancer therapy | FACT-G | 1(1.6) |
| HRQoL | EQ-5D | 1(1.6) | Medication adherence | MMAS-8 | 1(1.6) |
|  | EQ-5D plus EQ-VAS | 1(1.6) | Negative emotions | MCMQ | 1(1.6) |
| Physical wellbeing | FACT-Hep | 1(1.6) | Cognitive function | MMSE | 1(1.6) |
| Note:  PROs, Patient-Reported Outcomes; PROMs, Patient-Reported Outcomes Measures; QoL, Quality of Life; HRQoL, Health-Related Quality of Life; SCA, Self-Care Agency; EORTC QLQ-C30, European Organization for Research and Treatment of Cancer Quality of Life Questionnaire; SF-36, Short Form-36 Health Survey; FACT-Hep, Functional Assessment of Cancer Therapy-Hepatobiliary; FACT-G, Functional Assessment of Cancer Therapy-General; QoL-LC, Quality Of Life-Liver; EORTC QLQ-HCC18, European Organization for Research and Treatment of Cancer Quality of Life Questionnaire-Hepatocellular Carcinoma 18; WHOQOL-BREF, World Health Organization Quality of Life-Brief Version; QLACS, Quality of Life in Adult Cancer Survivors; GQOLI-74, Generic Quality of Life Inventory-74; EQ-5D, EuroQol Five Dimensions Questionnaire; EQ-VAS, EuroQol Visual Analogue Scale; PSQI, Pittsburgh sleep quality index; DBAS, Dysfunctional Beliefs and Attitudes on Sleep; VAS, Visual Analogue Scale; NRS, Numerical Rating Scale; SAS, Self-Rating Anxiety Scale; SDS, Self-rating depression scale; ESCA, Self-Care Ability Scale; DBAS, Dysfunctional Beliefs and Attitudes on Sleep; DLQI, Dermatology Life Quality Index; HF-QoL, Hand-Foot Skin Reaction and Quality of Life Questionnaire; SUPPH, strategies used by people to promote health; IWB, index of well-being; PDI, Patient Dignity Inventory; MMAS-8, Morisky Medication Adherence Scale-8; MCMQ, Medical Coping Modes Questionnaire; MMSE, Minimum Mental State Examination. | | | | | |

Table 3. The reporting quality of PRO in the included studies according to the CONSORT 2010 and CONSORT-PRO statement (*N* = 64)

| CONSORT 2010 and CONSORT-PRO item^a^ | | Complete report | Not complete report | No report |
| --- | --- | --- | --- | --- |
|  |  | *N*(%) | *N*(%) | *N*(%) |
| 1a | Identification as a randomised trial | 10(15.6) | 32(50.0) | 22(34.4) |
| 1b | Structured summary of the trial design, methods, results, and conclusions | 60(93.8) | 2(3.1) | 2(3.1) |
| **P1b** | **The PRO should be identified in the abstract as a primary or secondary outcome** | 5(7.8) | 49(76.6) | 10(15.6) |
| **2a** | **Scientific background and explanation of rationale** | 12(18.8) | 51(79.7) | 1(1.6) |
| 2b | Specific objectives or hypotheses | 55(85.9) | 8(12.5) | 1(1.6) |
| **P2b** | **The PRO hypothesis should be stated and relevant domains identified, if applicable** | 6(9.4) | 33(51.6) | 25(39.1) |
| 3a | Description of trial design (such as parallel, factorial) including allocation ratio | 62(96.9) | 1(1.6) | 1(1.6) |
| 3b | Important changes to methods after trial commencement (such as eligibility criteria), with reasons | 3(4.7) | 0 | 61(95.3) |
| 4a | Eligibility criteria for participants | 64(100.0) | 0 | 0 |
| 4b | Settings and locations where the data were collected | 56(87.5) | 5(7.8) | 3(4.7) |
| 5 | The interventions for each group with sufficient details to allow replication, including how and when they were actually administered | 21(32.8) | 42(65.6) | 1(1.6) |
| 6a | Completely defined prespecified primary and secondary outcome measures, including how and when they were assessed | 31(48.4) | 33(51.6) | 0 |
| **P6a** | **Evidence of PRO instrument validity and reliability should be provided or cited if available, including the person completing the PRO and methods of data collection (paper telephone electronic other)** | 6(9.4) | 48(75.0) | 10(15.6) |
| 6b | Any changes to trial outcomes after the trial commenced, with reasons | 2(3.1) | 0 | 62(96.9) |
| **7a** | **How sample size was determined** | 14(21.9) | 1(1.6) | 49(76.6) |
| 7b | When applicable, explanation of any interim analyses and stopping guidelines | 8(12.5) | 2(3.1) | 54(84.4) |
| 8a | Method used to generate the random allocation sequence | 44(68.8) | 3(4.7) | 17(26.6) |
| 8b | Type of randomisation; details of any restriction (such as blocking and block size) | 10(15.6) | 6(9.4) | 48(75.0) |
| 9 | Mechanism used to implement the random allocation sequence (such as sequentially numbered containers), describing any steps taken to conceal the sequence until interventions were assigned | 9(14.1) | 3(4.7) | 52(81.3) |
| 10 | Who generated the random allocation sequence, who enrolled participants, and who assigned participants to interventions | 10(15.6) | 1(1.6） | 53(82.8) |
| 11a | If done, who was blinded after assignment to interventions (for example, participants, care providers, those assessing outcomes) and how | 7(10.9) | 1(1.6） | 56(87.5) |
| 11b | If relevant, description of the similarity of interventions | 16(25.0) | 41(64.1) | 7(10.9) |
| 12a | Statistical methods used to compare groups for primary and secondary outcomes | 13(20.3) | 50(78.1) | 1(1.6) |
| 12b | Methods for additional analyses, such as subgroup analyses and adjusted analyses | 11(17.2) | 0 | 53(82.8) |
| **P12a** | **Statistical approaches for dealing with missing data are explicitly stated** | 5(7.8) | 0 | 59(92.2) |
| **13a** | **For each group, the numbers of participants who were randomly assigned, received intended treatment, and were analyzed for the primary outcome** | 55(85.9) | 8(12.5) | 1(1.6) |
| 13b | For each group, losses and exclusions after randomisation, together with reasons | 13(20.3) | 0 | 51(79.7) |
| 14a | Dates defining the periods of recruitment and follow-up | 10(15.6) | 13(20.3) | 41(64.1) |
| 14b | Why the trial ended or was stopped | 6(9.4) | 0 | 58(90.6) |
| **15** | **A table showing baseline demographic and clinical characteristics for each group** | 7(10.9) | 40(62.5) | 17(26.6) |
| **16** | **For each group, number of participants (denominator) included in each analysis and whether the analysis was by original assigned groups** | 59(92.2) | 0 | 5(7.8) |
| **17a** | **For each primary and secondary outcome, results for each group, and the estimated effect size and its precision (such as 95% confidence interval)** | 9(14.1) | 54(84.4) | 1(1.6) |
| 17b | For binary outcomes, presentation of both absolute and relative effect sizes is recommended | 9(14.1) | 10(15.6) | 45(70.3) |
| **18** | **Results of any other analyses performed, including subgroup analyses and adjusted analyses, distinguishing pre-specified from exploratory** | 13(20.3) | 0 | 51(79.7) |
| 19 | All important harms or unintended effects in each group | 35(54.7) | 4(6.3) | 25(39.1) |
| 20 | Trial limitations, addressing sources of potential bias, imprecision, and, if relevant, multiplicity of analyses | 21(32.8) | 4(6.3) | 39(60.9) |
| 21 | Generalizability (external validity, applicability) of the trial findings | 63(98.4) | 0 | 1(1.6) |
| **22** | **Interpretation consistent with results, balancing benefits and harms, and considering other relevant evidence** | 55(85.9) | 8(12.5) | 1(1.6) |
| **P20/21** | **PRO-specific limitations and implications for generalizability and clinical practice should be discussed** | 6(9.4) | 16(25.0) | 42(65.6) |
| 23 | Registration number and name of trial registry | 14(21.9) | 1(1.6) | 49(76.6) |
| 24 | Where the full trial protocol can be accessed, if available | 12(18.8) | 1(1.6) | 51(79.7) |
| 25 | Sources of funding and other support (such as supply of drugs), role of funders | 16(25.0) | 0 | 48(75.0) |
| Note:  PRO, patient-reported outcome; CONSORT, Consolidated Standards of Reporting Trials..  ^a^CONSORT PRO items are highlighted in bold. | | | | |

Table 4. CONSORT-PRO item adherence scores in the included studies (*N*=64)

| CONSORT-PRO item | RCT addressing the item (*N*=64) | | |
| --- | --- | --- | --- |
|  | *N* | % | Compliance rating |
| P1b. Abstract—PRO as primary/secondary endpoint |  |  |  |
| Item P1b completely addressed | 5 | 7.8 | poor |
| Item P1b partially addressed | 49 | 76.6 | Moderate |
| 2a. Rationale for including PRO endpoint | 24 | 37.5 | poor |
| P2bi. PRO hypothesis present | 11 | 17.2 | poor |
| P2bii. PRO domains in hypothesis | 6 | 9.4 | poor |
| P6ai. Evidence of PRO instrument validity | 33 | 51.6 | Moderate |
| P6aii. Statement of the person completing the PRO questionnaire | 15 | 23.4 | poor |
| P6aiii. Mode of administration (paper, e-PRO) | 6 | 9.4 | poor |
| P7a. How sample size was determined (not required unless PRO is a primary endpoint) | 0 | 0.0 | poor |
| P12a. Statistical approach for dealing with missing data (imputation, exclusion, other) | 5 | 7.8 | poor |
| 13ai. Report no. questionnaires submitted/available for analysis at baseline | 57 | 89.1 | good |
| 13aii. Report no. questionnaires submitted/available for analysis principle time point for analysis | 55 | 85.9 | good |
| 15. Demographics table includes baseline PRO | 47 | 73.4 | Moderate |
| 16. Number of pts (denominator) included in each PRO analysis | 59 | 92.2 | good |
| 17ai. PRO results reported for the hypothesised domains and time point specified in the hypothesis-OR-reported for each domain of the PRO questionnaire if no PRO hypothesis provided | 50 | 78.1 | Moderate |
| 17aii. Results include confidence interval, effect size or some other estimate of precision | 15 | 23.4 | poor |
| 18. Results of any subgroup/adjusted/exploratory analyses | 13 | 20.3 | poor |
| P20. PRO study limitations | 6 | 9.4 | poor |
| P21. Implications of PRO results for generalizability, clinical practice | 16 | 25.0 | poor |
| 22. PROs interpreted in relation to clinical outcomes | 55 | 85.9 | good |
| Note:  CONSORT, Consolidated Standards of Reporting Trials; PRO, Patient-Reported Outcome; RCT, randomized controlled trail; N/A, not applicable.  Compliance rating cut-off scores: “good” = >80% of RCTs within the group addressed the item; “moderate” = 50–79% of RCTs within the group addressed the item; “poor” = ≤49% RCTs within the group addressed the item. | | | |
